# An experimental study of the effect of neuromuscular blockade on EEG-based measures of awareness

**DOI:** 10.1038/s41598-026-50911-6

**Published:** 2026-05-02

**Authors:** Sebastian Halder, Bjørn E. Juel, Kenneth J. Pope, Andrew Hardy, John O. Willoughby, Johan F. Storm

**Affiliations:** 1 School of Computer Science and Electronic Engineering, University of Essex, Colchester, CO4 3SQ UK; 2 Vestre Viken Health Trust, Kongsberg Hospital, Kongsberg, 3612 Norway; 3 School of Informatics and Engineering, Flinders University, GPO Box 2100, Adelaide, 5001 SA Australia; 4 Department of Anaesthesia and Pain Management, Flinders Medical Centre, Bedford Park, 5042 SA Australia; 5 Department of Medicine, Flinders University, GPO Box 2100, Adelaide, 5001 SA Australia; 6 Brain Signalling Group, Section for Physiology, Department of Molecular Medicine, IMB, University of Oslo, Oslo, 0317 Norway

**Keywords:** Neuromuscular blocking agent (NMBA), Electroencephalography (EEG), Consciousness, Awareness, Accidental awareness during anaesthesia (AAGA), Medical research, Neurology, Neuroscience

## Abstract

Both basic and clinical consciousness research aims to find objective measures that reliably distinguish conscious from unconscious brain states. Electroencephalogram (EEG) measures are widely used, although they may be affected by interference from electrical signals such as those generated by muscles. To assess this source of error, we investigated the impact of neuromuscular blockade (NMB) on proposed measures of awareness (spectral slope, Lempel–Ziv complexity (LZc), connectivity, alpha peak frequency, power in canonical EEG frequency bands) computed from spontaneous high-density EEG recorded from six healthy volunteers in three different conditions: (1) awake-unparalysed (normal wakefulness), (2) awake-paralysed (complete paralysis caused by neuromuscular blocking agent (NMBA)), and (3) sedated-paralysed (deep sedation with propofol, with paralysis by NMBA). The measures we investigated distinguished awake-unparalysed states from sedated-paralysed with close to perfect accuracy in accordance with past findings. However, our analysis revealed a serious failure of most measures to recognise the awake-paralysed condition as an aware state. Errors ranged from 7% of awake-paralysed time segments predicted as unaware (using alpha power) to 100% (using LZc). Using a unique high-density EEG data set, this study clearly demonstrates that many EEG-based measures fail to recognise awareness in awake subjects under the influence of muscle relaxants. These results highlight critical limitations of current EEG-based measures at detecting awareness.

## Introduction

The widespread assumption that electroencephalogram (EEG) measures of consciousness are independent of muscular activity remains to be thoroughly tested under controlled conditions of neuromuscular blockade. Conventionally, developing such measures involves comparing wakefulness with states of apparent unconsciousness, such as deep sleep or general anaesthesia^1^. However, this approach implicitly equates unresponsiveness with unconsciousness^2,3^. In particular, the paralysed state induced by neuromuscular blocking agents (NMBAs), in which a patient appears unresponsive, may be mistaken for unconsciousness^4^. Thus, an objective measure of conscious awareness must go beyond distinguishing responsive and awake states from unresponsive states that are often assumed to be unconscious. A crucial test would show the measure’s capacity to differentiate between a state that is conscious but unresponsive [such as accidental awareness during general anaesthesia 25 (AAGA)] and an unresponsive state that is most likely associated with reduced consciousness (such as successful general anaesthesia sufficient for surgery).

Neuromuscular blockade (NMB) is used in 62% of general anaesthetic procedures, for easing intubation and optimising patient positioning^5–7^. NMBAs often have a longer duration of effect than anaesthetics^8^, leading to the risk that patients experience AAGA. AAGA with explicit recall is estimated to affect 1 in *∼*900 patients^9^, while connected responsiveness can be detected via isolated forearm technique (IFT) more frequently in controlled tests, particularly in young adults and females^10^, though this typically lacks explicit recall and painful experiences. Nevertheless, AAGA can be highly traumatic^11^, and the capacity to objectively measure its presence in real-time would be useful. While anaesthesia monitors are sometimes believed to be suitable for this purpose, commonly used indices like bispectral index^12,13^ and GM Entropy monitors, explicitly incorporate changes in electromyogram (EMG) activity in their algorithm. While this might be beneficial for most general anaesthesia monitoring, it severely limits the utility for detecting AAGA in situations with NMB where EMG activity is intentionally suppressed^14–17^, susceptible to EMG contamination^18^, or superimposed on EEG recording with NMB^19^. Thus, for the purpose of detecting awareness specifically in paralysed patients, dependence on EMG signals represents a fundamental limitation for any method of awareness detection in paralysed patients.

A rigorous test of any proposed EEG measure of awareness therefore requires a design that investigates muscular state and conscious state independently. Comparing an awake-unparalysed participant with an awake-paralysed one isolates the contribution of muscle activity, since both are conscious but only one has intact EMG. Conversely, comparing the awake-paralysed state with a sedated-paralysed state isolates the contribution of awareness, since both lack muscle activity but differ in conscious state. Only a measure that remains stable across the first comparison while showing a robust difference in the second can be considered a genuine marker of awareness rather than a surrogate of EMG.

Over the past decades, hundreds of EEG-based measures have been proposed as “measures of consciousness”^20^. Gathering enough data from surgical patients to evaluate measures of awareness is challenging because AAGA rarely occurs and explicit recollection of the event may be impaired^9^. Thus, many measures have largely been tested in controlled laboratory environments, and it remains unclear whether they are sensitive to EEG changes induced by NMBA, i.e. to EMG contamination^21^. Thus, the reliability of these EEG-based measures in detecting awareness as opposed to unresponsiveness, remains questionable.

In this study, we aimed to characterise how different EEG-based measures respond during selective NMB, leaving subjects awake and aware but paralysed. We therefore quantified the differential responses of state-of-the-art measures (e.g. based on LZc^22^, periodic and aperiodic components of the power spectrum^23,24^, and functional connectivity^25^) during normal wakefulness, awake paralysis with NMBAs, and sedation (also with NMBAs paralysis). Ideally, these measures would remain stable between the awake-unparalysed and awake-paralysed states, both conscious conditions, while showing a clear difference between these awake states and the paralysed-sedated state. This hypothesis reflects the fundamental expectation that valid consciousness measures track awareness itself, independent of motor output or muscle activity. If these measures fail to make this distinction, we cannot consider them as measures of awareness without further qualification or contextualization. To test this hypothesis, we analysed high-density EMG recordings from six healthy volunteers across three carefully contrasted conditions: awake-unparalysed, awake-paralysed (cisatracurium, with laryngeal mask airway (LMA) in situ), and sedated-paralysed (propofol, with LMA in situ). We predicted that a valid measure of awareness, one that tracks conscious state rather than muscular activity, would show no significant change between the two awake conditions, which share the same conscious state but differ in EMG, while showing a robust and consistent difference between either awake condition and the sedated-paralysed state. Failure to satisfy this criterion, specifically misclassification of the awake-paralysed state as unaware, would constitute direct evidence that the measure is driven by EMG rather than awareness itself.

## Results

For a measure to potentially detect awareness during paralysis, it should show no significant difference between the two ’aware’ states (awake-unparalysed and awake-paralysed), but a clear difference between the ’aware’ states and the sedated-paralysed state (assuming it is in fact a state of unawareness). This pattern would indicate that the measure may track awareness itself, rather than muscle activity (which is typically reduced in canonical states of “unawareness” like sleep and deep sedation, even when NMB is not present). Thus, in terms of classification of individual epochs the machine learning model should label awake-unparalysed and awake-paralysed epochs as ’aware’ and label epochs recorded during sedated-paralysed as ’unaware’. With the caveat that unconsciousness could not be objectively verified in this state in our study, we used this term as target label of the machine learning model.

### Effects of paralysis and sedation on power spectrum

**Fig. 1:**
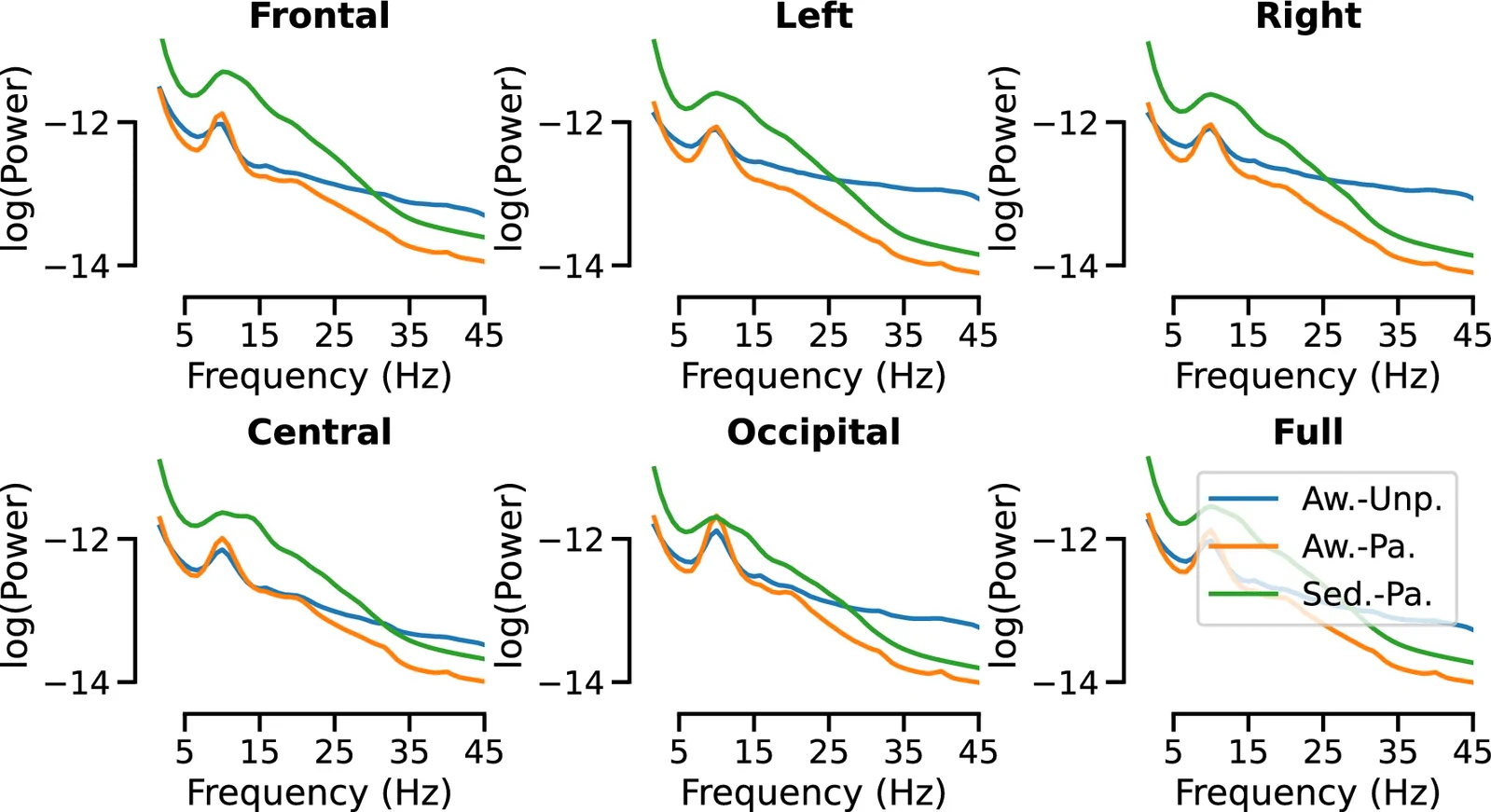
Power spectral densities visualised as line plots and averaged across all participants, epochs and channels. The blue lines represent awake-unparalysed, the orange awake-paralysed and green sedated-paralysed. Regions of interest were visualised individually using separate lines per condition. The final plot shows the power spectrum computed using the full channel set. The changes between conditions varied depending on the frequency range and scalp location. For example, compared to normal wakefulness, gamma power decreased the most in peripheral locations, e.g. by 7.4% in the left ROI (from −12.9 in the awake-unparalysed to −13.9 in the awake-paralysed state). For comparison, the decrease in the same band in the central ROI was only 3.3% from awake-unparalysed to awake-paralysed. Meanwhile, the biggest increases in power were seen in the alpha range, from awake-unparalysed to sedated-paralysed, e.g. increasing by 7.9% in the frontal ROI (from −12.3 in awake-unparalysed to −11.4 in sedated-paralysed). The occipital ROI showed the smallest changes across the full range between all conditions, although the gamma power appeared to decrease from the awake-unparalysed to awake-paralysed state, and sedation led to a general steepening of the spectrum and less extreme alpha peak.

Visual inspection of the group-averaged power spectra revealed that the awake-unparalysed and awake-paralysed conditions were highly similar below 15 Hz, with both states showing prominent oscillatory activity in the alpha range distributed across the scalp. In contrast, the sedated-paralysed state was characterised by a marked increase in overall power, particularly in the alpha band, which also appeared broader and more pronounced. Both the sedated and paralysed state showed a notable reduction in high-frequency (>15 Hz) power compared to the awake-unparalysed conditions. See Fig. 1.

### Effect of paralysis on measures of awareness

**Fig. 2:**
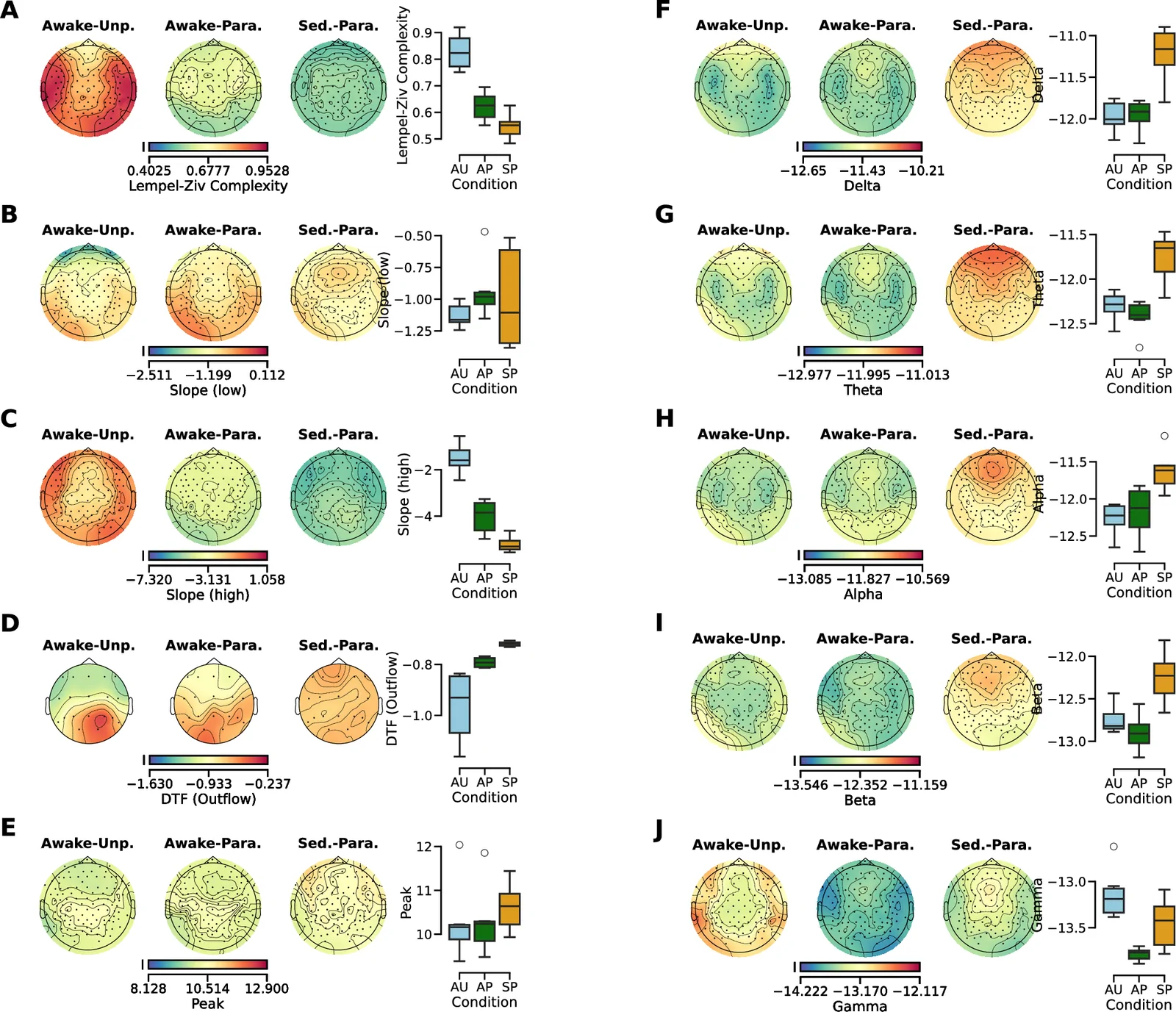
Difference of Lempel–Ziv complexity (**A**) and slope low band exponent (**B**), slope high band exponent (**C**), DTF outflow (**D**), alpha peak (**E**), delta (**F**), theta (**G**), alpha (**H**), beta (**I**) and gamma (**J**) power between awake-unparalysed, awake-paralysed and sedated-paralysed. The measures were visualised as the raw measure amplitude at each electrode location as a scalp topography (left, grand-averaged across all epochs and participants per condition) and as a distribution of values for each condition (the median is indicated as a black line; outliers, defined as values outside 1.5 times the interquartile range, are marked with circles). Values in the distribution plots were aggregated across all channels and epochs resulting in one value per participant and condition (six values per condition). For a measure to be considered successful, it should remain unchanged between awake-unparalysed (AU) and awake-paralysed states and only differ when comparing either awake state (AU/AP) to the sedated-paralysed (SP) state (e.g. delta power). For a measure to be considered misleading, AU must differ from AP and AP must not differ from SP (e.g. LZc).

When visually inspecting Fig. 2 (see Tables S1 and S2 for details) we observed awake-paralysed differed profoundly from awake-unparalysed for diversity, slope (high) and gamma, indicating dependence on EMG and they may not strictly be measures of awareness.

**Fig. 3:**
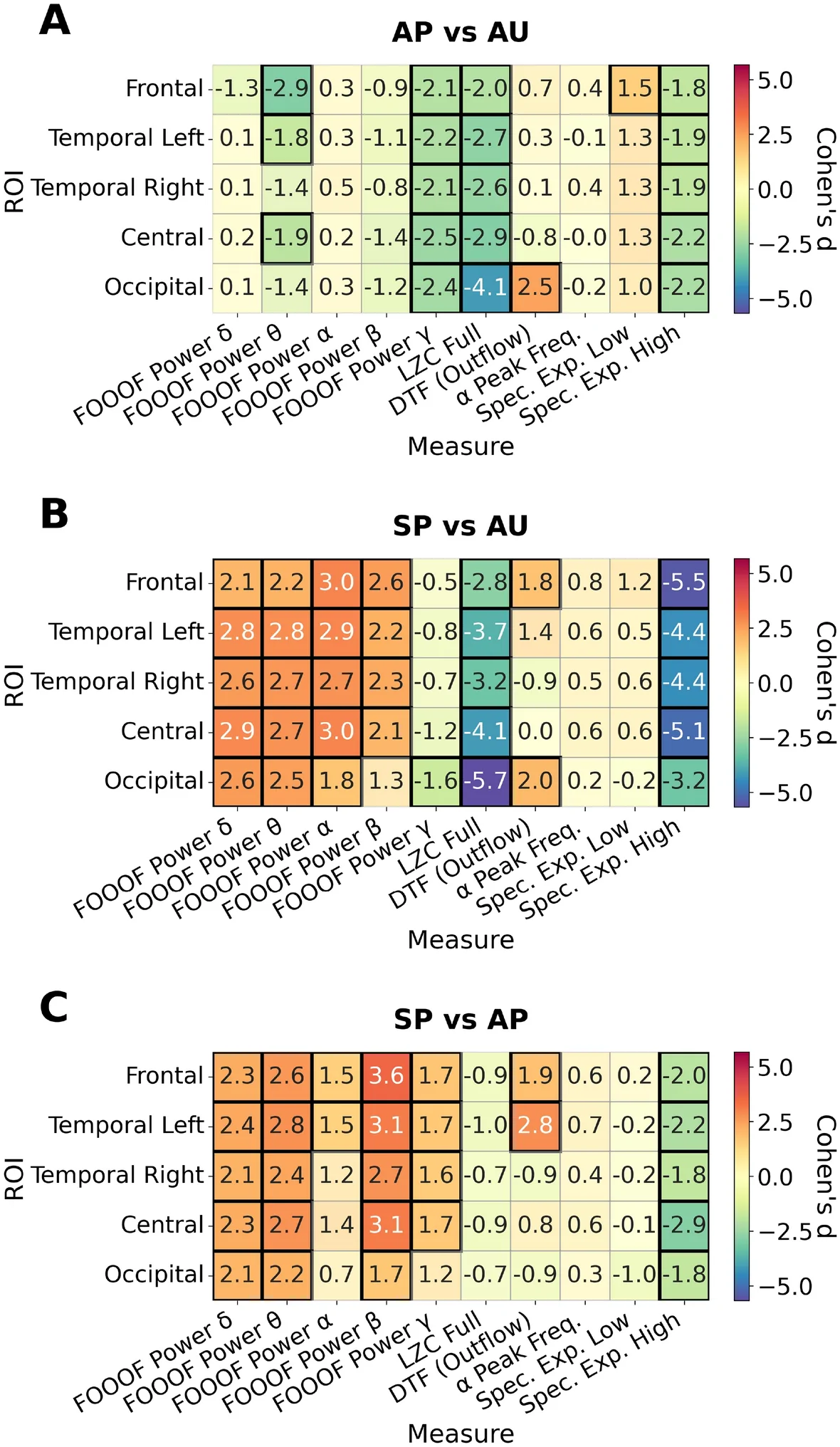
Effect sizes (Cohen’s d) of neuromuscular blockade on EEG-derived measures of awareness across brain regions. Heatmaps display Cohen’s d for paired samples (mean difference divided by the standard deviation of the differences) for ten EEG-based measures of awareness across five regions of interest (ROIs) for three condition comparisons: (**A**) Awake-Paralysed vs Awake-Unparalysed (AP vs AU), (**B**) Sedated-Paralysed vs Awake-Unparalysed (SP vs AU), and (**C**) Sedated-Paralysed vs Awake-Paralysed (SP vs AP). Statistical significance was assessed using paired t-tests with Benjamini-Hochberg false discovery rate (FDR) correction across all ROI-measure-comparison combinations; significant effects (p <.05, corrected) are outlined with thick black borders, whilst non-significant effects are shown with grey borders and a semi-transparent overlay. The colour scale represents standardised effect sizes, with positive values (red) indicating higher values in the first-named condition and negative values (blue) indicating higher values in the second-named condition. E.g. in panel **A**, in the column for Lempel–Ziv complexity (LZc), blue, negative values indicated higher values during AU than AP.

In Fig. 3A (comparing awake-paralysed with awake-unparalysed; thus a comparison of two awake states in which we would expect no differences for a measure of awareness), we observed significant effects in all region of interests (ROIs) for the gamma band, LZc and the spectral exponent (high). Theta power changed in the frontal, temporal left and central, spectral exponent in the frontal, and directed transfer function (DTF) on the occipital ROIs. The comparison of the two awake states further resulted in significant differences when computing values across all values and epochs for LZc (d = 2.1, t(5) = 6.62, p =.007) and spectral exponent high (d = 2.6, t(5) = −4.73, p =.031; Table S2).

In Fig. 3B (comparing awake-unparalysed with sedated-paralysed; thus a comparison of an aware state with a sedated state in which we expect differences for a measure of awareness; but the difference may be due the NMB) we found significant effects in at least one ROI for all measures except alpha peak frequency and the low component of the spectral exponent. It is worth noting the difference was inverted for theta.

Finally, in Fig. 3C (comparing awake-paralysed with sedated-paralysed; thus a comparison of an aware state with a sedated state in which we expect differences for a measure of awareness; in this case the difference can not be due the NMB as it was present in both states) we observed significant effects for all bands, frontal and left DTF and the high component of the spectral exponent. Unfortunately, we also observed significant misleading differences between awake-unparalysed and awake-paralysed (in both cases the participant was aware thus the measures of awareness should be identical) for the spectral exponent, theta and gamma power and DTF (Fig. 3A). Regarding theta, we can observe that the change from awake-unparalysed to awake-paralysed is in the opposite direction as the change from awake-unparalysed to sedated-paralysed (see Fig. 2G). Thus, based on these qualitative observations, raw delta, theta, alpha and beta power may be potential measures of awareness.

### Prediction of participant state

We then predicted the state of the participants from 5 sec epochs using a support vector classifier (SVC) in a leave-one-participant out cross-validation approach. This adds the temporal and spatial dimension that was not considered in the statistical analysis.

#### Naïve: training and testing on awake-unparalysed and sedated-paralysed

**Fig. 4:**
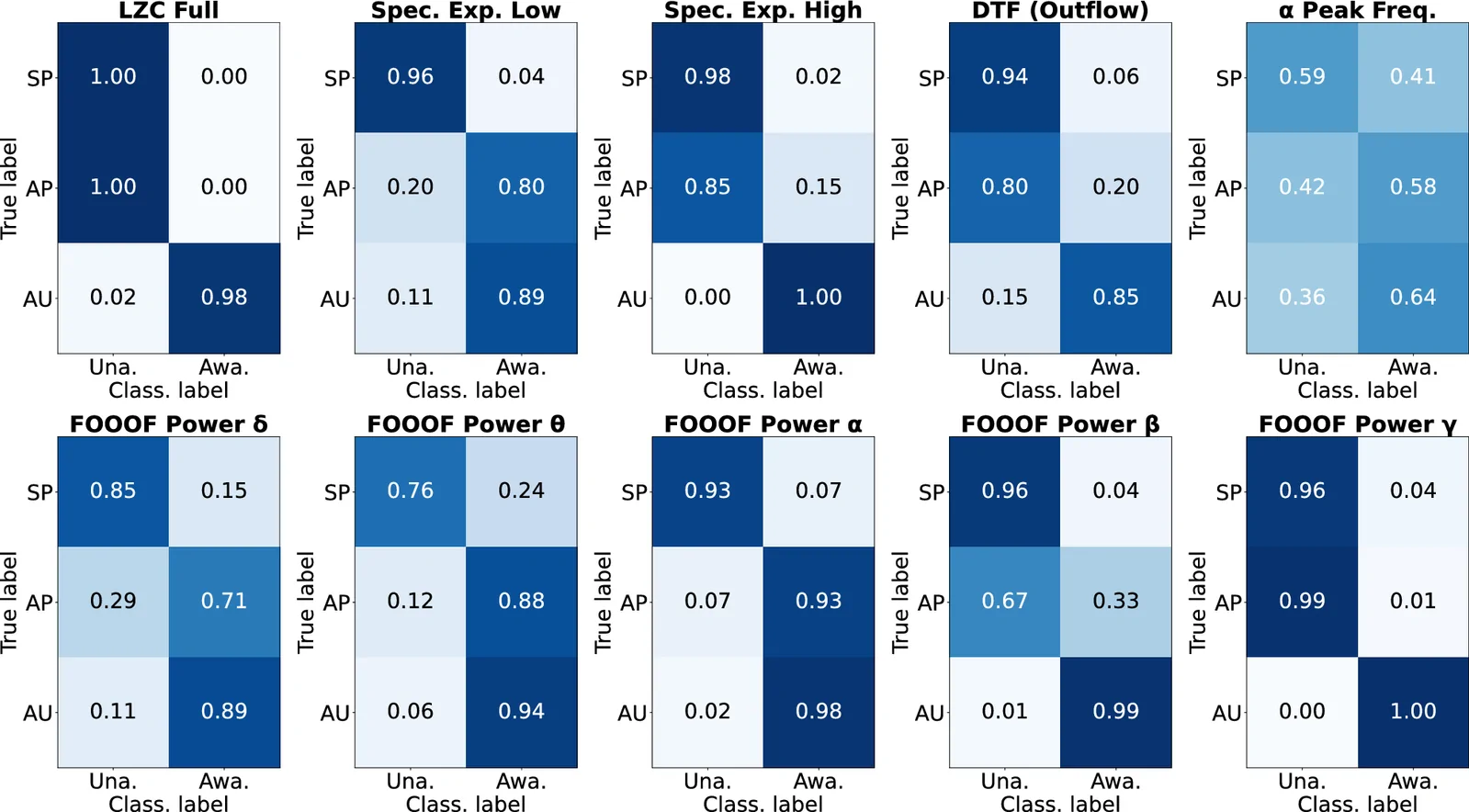
Confusion matrices that were computed by classifying individual epochs in a leave-one participant out cross-validation. The values reported in the matrix were computed as the mean across all cross-validation steps. The awake-paralysed (AP) state was included in testing with the same label as the awake-unparalysed (AU) state (two class approach, *3× 2* matrices). AP and AU trials predictions were rated as true when the prediction label was aware (awa.). Sedated-paralysed (SP) trials were rated as true if labelled as unaware (una.). Results were computed using the measures indicated in the title of each matrix. For a measure to be considered successful, it should classify both the awake-unparalysed and awake-paralysed states as aware, and the sedated-paralysed state as unaware. Considering SP and AU trials, there was near perfect prediction using diversity, slope (high), beta and gamma power. Prediction using peak alpha frequency was poor. Viewing the accuracy for the AP trials, revealed most measures predicted the awake-paralysed state as unaware. The measure that achieved the highest accuracy in this task was alpha power.

Following the standard approach of comparing the awake-unparalysed and sedated-paralysed states, i.e. excluding the awake-paralysed state, led to near perfect prediction using diversity, slope (high), beta and gamma power (Fig. 4). Prediction of the sedated-paralysed state using outflow and slope (low) was also high (96% and 94% respectively) but both methods gave >10% error predicting the awake-unparalysed state. Prediction using delta, theta and alpha power was higher for the awake-unparalysed state (89%, 94% and 98%) than the sedated-paralysed state (85%, 76% and 93%). Prediction using peak alpha frequency was poor for both sedated-paralysed and awake-unparalysed states—59% and 64% respectively. See Fig. 4 rows for sedated-paralysed and and awake-unparalysed. This is the comparison that corresponds to Fig. 3B.

#### Two classes: training on awake-unparalysed and sedated-paralysed and testing on all three states

The question we wanted to answer using this approach was whether a classifier trained on the data from awake-unparalysed and sedated-paralysed states (data that is available in abundance, and is commonly used for testing measures of consciousness) would predict the awake-paralysed state as unaware or aware. Predicting awake-paralysed as aware would mean the measure might conceivably detect awareness without being confounded by muscle activity—a minimal requirement for any successful measure according to our assessment. This is more likely to be successful if there are no differences between the features in Fig. 3A and large differences in Fig. 3C that have the same orientation as differences in Fig. 3B.

The highest accuracy was achieved using alpha power (93% of awake-paralysed epochs correctly predicted as aware, 95% CI [0.44–0.97]). All other measures were either close to random (alpha peak frequency) or biased towards predicting awake-paralysed as unaware. For example, LZc and gamma power classified 0% (95% CI [0.00–0.39]) and 1% (95% CI [0.00–0.39]) of awake-paralysed epochs as aware respectively (Table S3). Also see confusion matrices in Fig. 4 row for awake-paralysed condition.

#### Exploratory analysis of 55 features

**Fig. 5:**
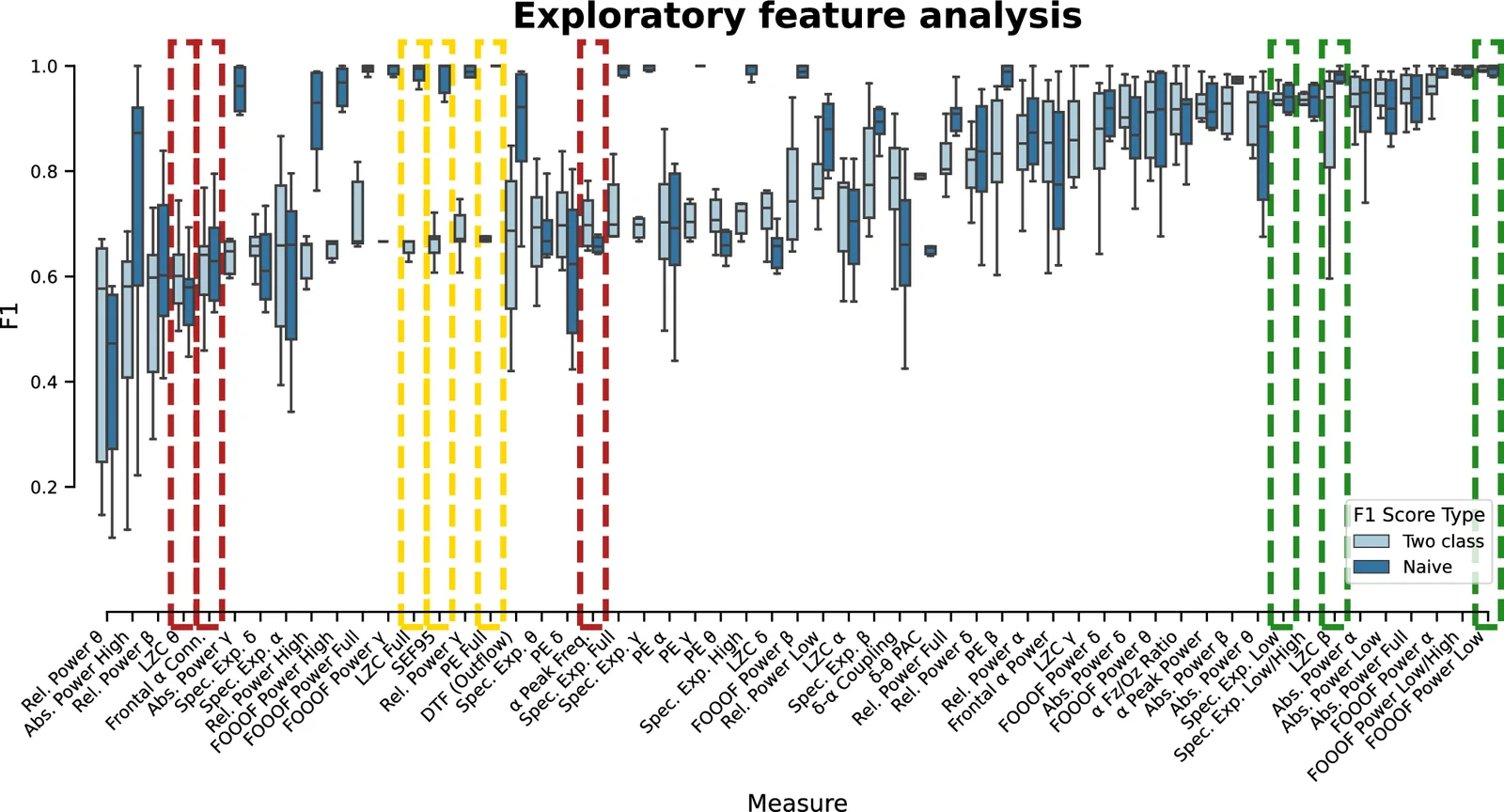
Exploratory feature analysis of classification performance. Box plots show the distribution of F1 scores for various electroencephalogram (EEG) derived measures used to classify states. F1 scores are differentiated according to training protocol: naive (dark blue, awake-paralysed (AP) state was excluded from training and testing) and two class (light blue, AP excluded from training and included in testing with the same label as the awake-unparalysed (AU) state; they can be considered as a measure of success in the context of our investigation and the measures are sorted from right to left according to success). We highlighted nine exemplary measures with a coloured dashed box. Green: reliably predict awake-paralysed as awake, yellow: reliably predict sedated-paralysed as distinct from awake-unparalysed but confuse with awake-paralysed; red: confuse all three states.

The performance of each measure was quantified using the $F_1$ score (see Fig. 5) both for the naïve and the two-class approach. The highest performance was achieved with fitting oscillations & one-over-F (FOOOF) features averaged across the 1–20 Hz band, with FOOOF Low/High ratio and FOOOF Low both achieving a median two-class $F_1$ of 0.99 and overall accuracy of 0.95 (95% CI [0.74–0.99]) and 0.93 (95% CI [0.67–0.97]) respectively (Table S3). Interestingly, LZc restricted to the beta band performed quite well according to the median (two-class $F_1$ = 0.94; naïve $F_1$ = 0.98) but the distribution was wider than for other measures, reflected in a broader AP accuracy confidence interval of 0.64 (95% CI [0.19–0.81]; Table S3). LZc (full band), permutation entropy (full band) and spectral edge frequency 95 appeared to be strongly influenced by muscle activity, correctly classifying all SP epochs (SP accuracy = 1.00) while failing to detect awareness in AP epochs (AP accuracy: 0.00, 0.03, and 0.04 respectively; 95% CI [0.00–0.39] for all three; Table S3). Several measures were unable to distinguish any of the states, e.g. LZc restricted to the theta band, frontal alpha connectivity, and alpha peak frequency, with overall accuracies of 0.55 (95% CI [0.29–0.71]), 0.57 (95% CI [0.34–0.75]), and 0.60 (95% CI [0.34–0.75]) respectively, and two-class $F_1$ scores of 0.60, 0.64, and 0.70 (Table S3).

## Discussion

Our findings revealed that while many EEG-based measures could readily distinguish awake-unparalysed from sedated-paralysed, they failed to recognise awareness in the awake state with paralysis. Notably, alpha power was the most accurate measure for predicting awareness using five second epochs in the paralysed wakefulness state. This work highlights the remarkably strong impact of muscle activity on current EEG-based measures of awareness, and indicates they may be measures of a common confound (EMG activity) rather than awareness.

The propofol doses used in this study, particularly for participant 0, were more consistent with deep sedation than with surgical anaesthesia. This was intentional, as outlined in “Experimental Methods”. While unconsciousness may occur at these concentrations in healthy volunteers^26,27^ we acknowledge the limitation that, due to the ischaemic limit of the IFT, we could not objectively verify unresponsiveness during the sedated-paralysed state. Although all 6 participants afterwards recalled no experience (including train-of-four (TOF) testing) from the propofol sedation period, retrospective participant reports may be confounded by the amnestic effects of propofol (as always in studies of consciousness involving sedatives). Hence, our findings regarding the effects of propofol combined with NMB (sedated-paralysed) on EEG measures should be interpreted as reflecting deep sedation rather than definitive surgical anaesthesia. Furthermore, although the sample size of six participants is small, the statistical power of the primary findings is sufficient to support the main conclusions. For measures that failed to detect awareness under paralysis, effect sizes were large and consistent across all participants without exception (e.g., LZc: d = 2.1; spectral exponent, high component: d = 2.6). For measures with smaller effect sizes, such as DTF outflow (d = 0.5) and the low component of the spectral exponent (d = 0.4), the present sample size limits definitive interpretation and replication in larger cohorts is warranted.

The measures we investigated in this study were computed from the spontaneous EEG and can thus be utilised for time resolved monitoring. The implications for their capacity to detect AAGA are apparent from the tests including the paralysis condition in the test set (Fig. 2 and 4 row for awake-paralysed). Here we observed an increased bias towards categorising the awake-paralysed state as unaware, with the exception of features based on alpha power. For example, LZc over all electrodes changed from awake-unparalysed 0.84, awake-paralysed 0.62, to 0.55 sedated-paralysed whereas alpha log(power) over all electrodes changed from normal −12.27, paralysis −12.2, to −11.63 anaesthesia. This effect was consistent over all participants. In Fig. S3 we observed an increase in LZc between awake-paralysed and sedated-paralysed states in all participants. This was also reflected in the classification of individual epochs using the SVC. Using LZc as the only feature, 0% of awake-paralysed epochs were correctly classified as aware. In contrast, using alpha alone resulted in 93% of the paralysed trials being classified as aware.

The anteriorisation of alpha induced by propofol is a commonly observed phenomenon^28,29^. Unfortunately, decreases in alpha have also been observed^30^. The impact on alpha has also been reported to be drug dependent: 4% of patients anaesthetised with sevoflurane or desflurane did not exhibit any alpha^31^. The effect of Xenon on alpha appears to be inconsistent^32,33^. Ketamine appears to cause changes in alpha peak frequency^23^. The diagnostic value of alpha power for detecting awareness in patients with disorder of consciousness appears to depend on etiology^34^. Thus, a further limitation of our study is that it is unlikely that alpha log(power) individually is a reliable marker of awareness for all anaesthetic agents but may play a crucial role if the field moves from trusting indices to interpreting spectrograms^35^.

A further source of variability inherent to this study is inter-individual heterogeneity in neuroanatomy and cortical geometry, which is known to affect scalp-recorded EEG amplitude and topographic distribution^36^. Additionally, acquisition-related factors, including impedance variability across sessions, electrode placement, and minor differences in cap positioning^37^ may introduce noise that could limit the general applicability of measures relying heavily on alpha amplitude alone. Given the small sample of six participants, such sources of variability could disproportionately influence group-level estimates and should be considered when interpreting the generalisability of the findings.

EEG has been used to study effects of anaesthetic agents on brain activity for more than 85 years^38,39^. However, it has proven difficult to find an EEG-based monitor capable of reliably detecting AAGA. One reason for this may be EMG overlapping substantially with the EEG signals^21^, which would explain the unreliability of the widely used commercial indices^15–17^ that explicitly use EMG in their algorithms. In prior work, while developing our own putative DTF-based marker of consciousness, we focused on slower (theta and alpha) frequencies of the EEG with the hope that this would avoid overlap with EMG^25,40^. However, having now investigated this directly we find that this marker is also influenced by muscle activity, leading to misclassification of aware vs. unaware states. Albeit more theoretically grounded, measures based on LZc also changed significantly between awake-unparalysed and awake-paralysed, and surprisingly there was no significant difference between awake-paralysed and sedated-paralysed (as reflected in classification with the SVC in Fig. 4). We observed the strongest decrease of LZc on peripheral electrodes, near major muscles, suggesting that the randomness measured with this method was also influenced by EMG. The sensitivity of the investigated EEG measures to EMG contamination can be partly understood through the spectral properties of the measures themselves. Broadband measures spanning high frequencies, which included the high component of the spectral exponent and LZc, were most strongly affected, consistent with the known spectral overlap between EMG and EEG above 30 Hz^21^ For the spectral exponent specifically, this overlap is particularly problematic because propofol independently produces a broadband spectral change^41^, meaning that EMG removal and sedation produce superficially identical high-frequency spectral signatures. Alpha power, by contrast, reflects a clearly discernible oscillatory peak in a frequency range largely below the EMG contamination band; spectral peaks of this kind are theoretically resistant to broadband contamination, whether of neural or muscular origin. The sensitivity of LZc to EMG likely reflects a more direct mechanism: high-frequency arrhythmic muscle bursts increase time-domain signal complexity, an effect that was spatially consistent with EMG source proximity in the present data (see Fig. 2A). In summary, although several measures can easily discriminate awake-unparalysed from sedated-paralysed, EMG contamination caused confusion of awake-paralysed with sedated-paralysed and remains a strong source of error for identifying awareness specifically. This error remained even when awake-paralysed state training data was provided, suggesting an overlap of the features between these conditions (see Supplemental material).

In addition to the use of spontaneous EEG, consciousness has been assessed by perturbational or task-based EEG measures.

The perturbational complexity index (PCI), developed by M. Massimini and colleagues^42^ has been thoroughly tested and seems highly reliable for distinguishing conscious from unconscious brain states, including wake vs. sleep and general anaesthesia, and is probably less influenced by EMG due to the perturbational approach it is founded on^43^. However, because PCI requires multiple (*∼*100) repeated stimuli (usually by transcranial magnetic stimulation) and averaging of EEG responses, it has a low time resolution, and is thus not so suitable for monitoring patients to rapidly detect awareness, e.g. to detect AAGA.

Responses to sensory input and event-related potentials (ERPs) can also indicate altered awareness^44,45^. Like PCI, these approaches require multiple trials, thus reducing time resolution.

Other approaches rely on active tasks, e.g. IFT, which does not require EEG or stimulation; but this can be used only for *∼*20 minutes due to the need for reperfusion of the ischaemic, isolated limb^4^. Also brain-computer interfaces can be used to by-pass the need for muscular control, e.g. using motor imagery^46,47^. Investigations of dosage and anaesthetic agents may be needed to understand the mechanisms of disconnection and find the optimal way to detect AAGA^48^.

In summary, alternative methods such as PCI and ERP^49^ are proabably less affected by EMG, but suffer from low temporal resolution, more complex setup, or limited duration (IFT).

In conclusion, we found that when the awake-paralysed state was included in the test set, many measures failed to correctly classify it as aware. We can thus only conclude that EMG has a surprisingly strong influence on EEG-based measures, in particular on LZc, and that the perfect measure of awareness has not yet been found. On our data, log(power) features in the alpha band achieved the highest accuracies (Fig. 4). Since different anaesthetics vary in their impact on the alpha rhythm, and there is no obvious theoretical grounding for why the alpha rhythm should be particularly important for awareness, it is not reasonable to generalise this observation. In an exploratory analysis we found the average power in the 1–20 Hz band computed with FOOOF to have the highest accuracy at classifying the awake-paralysed state as aware (Fig. 5) but this will also have to be verified with other anaesthetic agents. Our investigation highlights a critical limitation of relying on EEG recorded without muscle paralysis to develop measures of awareness, as EMG activity is absent by design during many surgeries and a true measure of awareness should not be affected by EMG activity alone. Alternative EEG measures, potentially relying on tasks^47,50^ or perturbation^42^, which are robust to the absence of EMG, are needed for reliable monitoring of awareness.

## Methods

The latest protocol for this experimental study was approved by the Southern Adelaide Clinical Human Research Ethics Committee (SAC HREC EC00188) on 24 February 2014, and written informed consent was obtained from all subjects. All methods were performed in accordance with the relevant guidelines and regulations.

### Data

EEG was recorded with a 115 channels Neuroscan EEG amplifier (Compumedics, Victoria, Australia), at 5000 Hz with a 1250 Hz low-pass filter, a 128-channel cap with Ag-AgCl electrodes (Easy Cap GmbH, Germany), reference placed on the left ear, impedances were below *5kΩ*, electrooculogram, electrocardiogram, respiration and EMG sensors. The sedation recordings, though collected alongside earlier work^49,51,52^, remained unanalysed until this study. The current authors initiated re-examination of the full dataset to address previously unexplored questions regarding effects of NMB on EEG signatures of consciousness.

### Experimental procedure

**Fig. 6:**
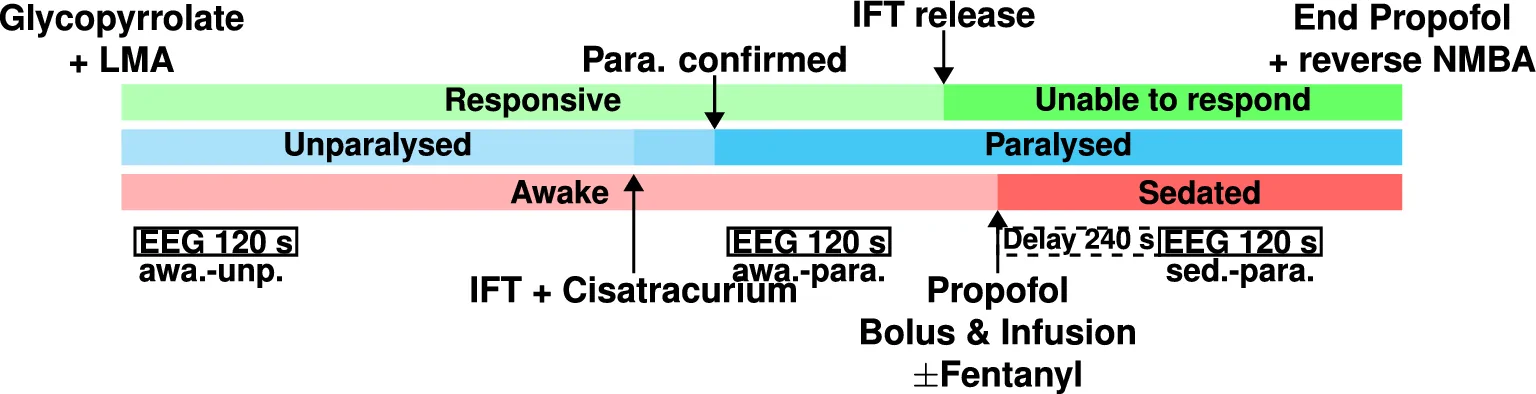
Timeline illustrating the sequence of key events and physiological states during the anaesthetic protocol with labels describing specific events (drug administrations, procedural steps, confirmations). Shaded rectangles indicate the occurrence of different states: ’Awake’ changed to ’Sedated’ after administration of propofol (red), ’Unparalysed’ changed to ’Paralysed’ after administration of the NMBA Cisatracurium (blue), ’Responsive’ changed to ’Unable to respond’ after IFT release (green). White boxes below each state rectangle indicate 120-second EEG segment used for the current analysis. The ’Sedated’ segment was extracted 240 seconds after the bolus. Below each box we indicate which designation we use for this state in the text (awa.-unp.: awake-unparalysed, awa.-para: awake-paralysed, sed-para.: sedated-paralysed). Due to a protocol deviation Neostigmine was administered before recording the sedation EEG for Participant 5. Note that the sizes of the rectangles are not proportional to the duration in seconds.

This study represents a re-analysis of EEG data originally collected between 2006 and 2008 for different research objectives. Initial participants were medically qualified volunteers associated with the research group, who were knowledgeable about the procedural risks and understood the significance of the research question; subsequent participants were recruited through interest in the laboratory’s research programme. Exclusion criteria included pre-existing cardiovascular or neurological disease (excluding migraine). Of the ten volunteers recruited (six male, four female), four (three female, one male) were unable to tolerate awake laryngeal mask airway insertion and did not complete the protocol. The remaining six participants (five male, one female), who completed all experimental procedures, form the study sample reported here. Spontaneous EEG was recorded from all volunteers in three states, always with with eyes closed (the term spontaneous EEG refers to activity recorded during rest, without any external stimulation or task). Due to the nature of the experiment the sample size was not based on a power analysis but on the availability of volunteers. Recordings took place in a Faraday cage in an EEG laboratory at Flinders University, Australia. Participants received a dose of glycopyrrolate (see Table 1 for all dosages) and the LMA was inserted (awake-unparalysed, see Fig. 6). Propofol sedation was administered as a necessary procedural requirement to ensure participant comfort and permit safe maintenance of the LMA during recovery from neuromuscular blockade in a non-theatre environment. This phase was not designed to achieve the depth of anaesthesia used in surgical practice; rather, the dose was titrated to tolerance of the LMA, consistent with deep sedation. The EEG recorded during this phase opportunistically provided a sedated-paralysed comparator condition for the present analysis. Total propofol dose was not documented in clinical records for Participant 0. Height was not recorded for any participants. The IFT^4^ was applied and NMBA was administered. Paralysis was confirmed via EMG (awake-paralysed). The IFT was deflated at the limit of ischaemic tolerance. From then on the participants were unable respond (and responsiveness could no longer be confirmed if present). Subsequently participants were sedated using Propofol. Propofol was administered using a Graseby 3500 Anaesthesia Pump incorporating Diprifusor TCI Version 2, which employs the Marsh pharmacokinetic model. All subjects received a machine-calculated bolus followed by an infusion titrated to maintain the target plasma concentration (see Table 1). The bolus doses were consistent with the Diprifusor used for deep sedation, not surgical anaesthesia, and each bolus was followed by a continuous infusion. The aim of the sedation was to maintain tolerance of the LMA in a non-theatre environment until the neuromuscular blockade could be safely reversed. Four participants received a dose of fentanyl at the time of the propofol bolus (Participants 3 and 5 were opioid intolerant). Reversal of neuromuscular blockade was initiated only after TOF monitoring indicated the onset of spontaneous recovery, ensuring that the paralysis state was maintained during EEG acquisition in the sedation phase. Due to a protocol deviation Neostigmine was administered before recording the sedated state for Participant 5. Participants were asked to perform different mental tasks during the awake-unparalysed and awake-paralysed state which were not analysed in the present work (see Witham et al., 2008^51^). We included step-by-step details of the protocol in Table 2.

**Table 1:** Summary of sedation and neuromuscular blockade protocol. The table includes subject demographics, propofol dosing parameters, and neuromuscular blockade management details. Target plasma concentration of propofol varied between experiments, with initial targets ranging from 2 mcg ml$^{-1}$ to 4 mcg ml$^{-1}$. Neostigmine (2.5 mg) was administered with either atropine (1.2 mg) or glycopyrrolate (0.4 mg) for reversal of neuromuscular blockade. Total propofol doses are reported in milligrams (mg), with “NS” indicating this was not stated in the records.

Part.	Age	Sex	Weight (kg)	Target (mcg ml$^{-1}$)	Bolus (mg)	Bolus (sec)	Infusion (mins)	Total (mg)	Fentanyl (mcg)	Reversal
0	63	M	80	2$^1$	37	10	45	NS	100	Neo. + Atrop.
1	42	M	105	Variable$^2$	40$^3$	14–21$^3$	40	1100	100	Neo. + Atrop.
2	73	M	95	4	87	26	30	550	100	Neo. + Atrop.
3	58	M	65	4	59	17	33	NS	0$^4$	Neo. + Atrop.
4	40	M	78	4	71	21	40	800	50	Neo. + Atrop.
5	47	F	68	4	62	18	22	200	0$^4$	Neo. + Atrop.

$^1$Participant 0 was persistently hypertensive during the sedation phase. $^2$Participant 1 had a variable and increasing target concentration during infusion due to clinical signs of inadequate sedation. $^3$Range reflects the bolus doses associated with each change of target concentration. $^4$Four participants received fentanyl, and two did not due to an expressed intolerance or unwillingness for opioids..

**Table 2:** Overview of the eight experimental phases in the experimental protocol, detailing preparation, paralysed-awake assessment with the isolated forearm technique, induction of neuromuscular blockade and ventilation, EEG recordings during paralysis and sedation, and subsequent neuromuscular recovery and emergence.

Phase	Description
Preliminary	Preparatory training of subjects to tolerate a LMA of appropriate size, using topical anaesthesia (4% lignocaine gel)
Phase 1	Room-sized Faraday cage. Subject seated with legs supported
	Two anaesthetists—Anaesthetist 1 inside and Anaesthetist 2 outside the Faraday cage. Anaesthetic communication by hand signals (CCTV). (Each anaesthetist had the same role in each experiment.)
	Monitoring: End Tidal CO_2_, ECG, Pulse Oximetry, and Peripheral Nerve Stimulation (simple Train of Four [TOF], right common peroneal nerve)
	All anaesthetic equipment except oximetry probe and peripheral nerve stimulator was outside the Faraday cage
	Glycopyrrolate 0.4 mg IV to minimise oral secretions
	Insertion of Laryngeal Mask Airway
	Spontaneous ventilation. Air/O_2_ mix by circle anaesthesia circuit
Phase 2	Pre-paralysis study—Resting EEG and cognitive tasks (the latter not analysed in this publication; see Witham et al.^49,51^)
Phase 3	IFT using non-dominant arm (150% resting BP)
	Cisatracurium 20 mg IV
	Establish IPPV to subjects’ comfort (hand signals)—fluid logic ventilator, rising bellows (Ulco Medical Pty Ltd)
	Confirm NMB by simple TOF
Phase 4	Paralysis study—resting EEG and cognitive tasks (not analysed in this publication)
	IFT (communication & tasks) up to 18 minutes
	IFT cuff deflation (ischaemic paralysis)
Phase 5	Sedation & ventilation until resolution of NMB
	Aims of sedation:
	1. Toleration of LMA & IPPV as topical airway anaesthesia wears off
	2. Cough suppression with fentanyl
	3. Sedation using Graseby 3500 Anaesthesia Pump, Incorporating Diprifusor™, TCI Version = 2. (Marsh model: bolus + infusion regime to a target plasma concentration.)
	Dose adjustments based on clinical observation of the subject
Phase 6	Sedated EEG recording—only published in this paper
Phase 7	Spontaneous resolution of NMB assessed by simple TOF twitch count
	Reversal of NMBA with neostigmine & atropine
	Stop propofol infusion
Phase 8	Awakening & extubation

### Pre-processing

The EEG data were pre-processed in two stages. In the first stage, conducted in 2018 using EEGLAB v14.1.1^53^, the raw Neuroscan.cnt recordings were imported, downsampled to 250 Hz, and re-referenced to REST^54^. These pre-processed files were stored and the EEG analysis reported here was not conducted until 2024. In the second stage, using MNE-Python v1.7^55^, the following steps were applied in the order listed:

1. High-pass filtering at 1 Hz
2. Extraction of a 120-second continuous segment per condition
3. Segmentation into 5-second epochs with 50% overlap (47 epochs per participant per condition).

### Feature extraction

We extracted five categories of features. (1) Logarithmic *bandpower* from the canonical frequency bands (delta 1–3.5.5 Hz, theta 3.5–8.5 Hz, alpha 8–13 Hz, beta 13–26 Hz, gamma 26–45 Hz) and the full band from 1–45 Hz. Power spectral density (PSD) was computed for each channel and epoch using the Welch method (resulting in 53 frequency bins). We then used FOOOF^24^ to parameterise the power spectra for each epoch and channel and stored the logarithmic bandpower for each frequency bin. (2) *Spectral slope* was computed using identical processing with the difference that we stored the exponent of the aperiodic component for each channel and epoch for each of the canonical bands, the full band (1–45 Hz), the low half (1–20 Hz) and the high half (20–40 Hz)^23^ using Python scripts kindly provided by the authors to compute the (negative) spectral exponent *β*. (3) *Peak frequency* was again computed using identical processing as (1) with the difference that we stored the frequency of the peak of each of the bands in Hz as determined with FOOOF. (4) *Signal diversity* was computed for each epoch and channel on the time domain signal using single channel LZc^22^ with Python scripts kindly provided by the original authors. (5) *DTF (Outflow)*^25^, was computed by estimating brain connectivity with DTF^56^ and computing the median of all outgoing connections for each electrode. We computed DTF between 8–12 Hz and re-referenced the data to FC1, for a subset of 19 EEG channels making the procedure as similar as possible to the original work, using the Python script provided by the author (also one of the co-authors of this paper). In an exploratory analysis we expanded our original selection of features to a total of 55 measures derived from the metrics Casey et al.^57^ investigated. See Section “Exploratory analysis of features” in the Supplemental Material for details.

### Data splitting and labelling

We evaluated how accurately we could predict the state of the participants using measures of awareness extracted from 5 sec EEG epochs using a linear SVC. This design corresponds to an update of the state of the patient every five seconds. Training and testing was performed following a leave-one-participant-out scheme (five of the participants were used for training and feature selection, the remaining participant was for testing). We labelled each epoch as either ’aware’ (awake-unparalysed and awake-paralysed) or ’unaware’ (sedated-paralysed; please note discussion above in that we could not objectively verify if the participants were unaware (or unconscious)). Training and testing used two sets of classes. (1) *Naïve:* the awake-paralysed data was excluded from training and testing. The “Naïve” approach resembles the standard way EEG measures of consciousness (including anesthesia monitors) are tested in the field of consciousness science—if they can reliably distinguish awake-unparalysed from sedated-paralysed, they are taken to work well—and we include it here to reproduce prior work. (2) *Two classes*: awake-paralysed data was excluded only from training but included in testing with the same label as the awake-unparalysed data (i.e. ’aware’). In the confusion matrices we indicate the true label of the awake-paralysed class resulting in a *2 × 3* matrix. “Two classes” was the crucial test in this paper—measures should reliably classify participants under the effect of NMBAs as aware to be considered measures of awareness (or consciousness).

### Model training and testing

Classification was performed using scikit-learn^58^. We applied a standard scaler, used a linear SVC for feature selection (L1 penalty), and a linear SVC for classification (C=1, gamma=’auto’).

We evaluated the accuracy of classification using confusion matrices and $F_{1}$ scores. The $F_{1}$ score was computed as the harmonic mean of precision and recall^59^. In the context of awareness detection we can view precision as the number of time segments in which a patient is aware that were classified as aware divided by the total number of time segments that were classified as aware. A high precision value means the classifier is mostly correct when predicting awareness but may miss times of awareness. Similarly we can view recall as number of aware time segments classified as aware divided by the total number of aware time segments. A high recall value means the classifier will not miss segments of awareness but may mistake segments of unawareness as aware. Since the $F_{1}$ score combines both metrics a high $F_{1}$ score means the classifier does not miss segments of awareness and does not mistake segments of unawareness for aware.

### Statistical analysis

Group-level statistical comparisons aggregated values across all channels and epochs within each participant, yielding one representative value per condition per participant for paired t-tests. We assessed statistical significance of the differences of the measures between states on the group level using paired t-tests with Bonferoni correction (Table S2).

**Fig. 7:**
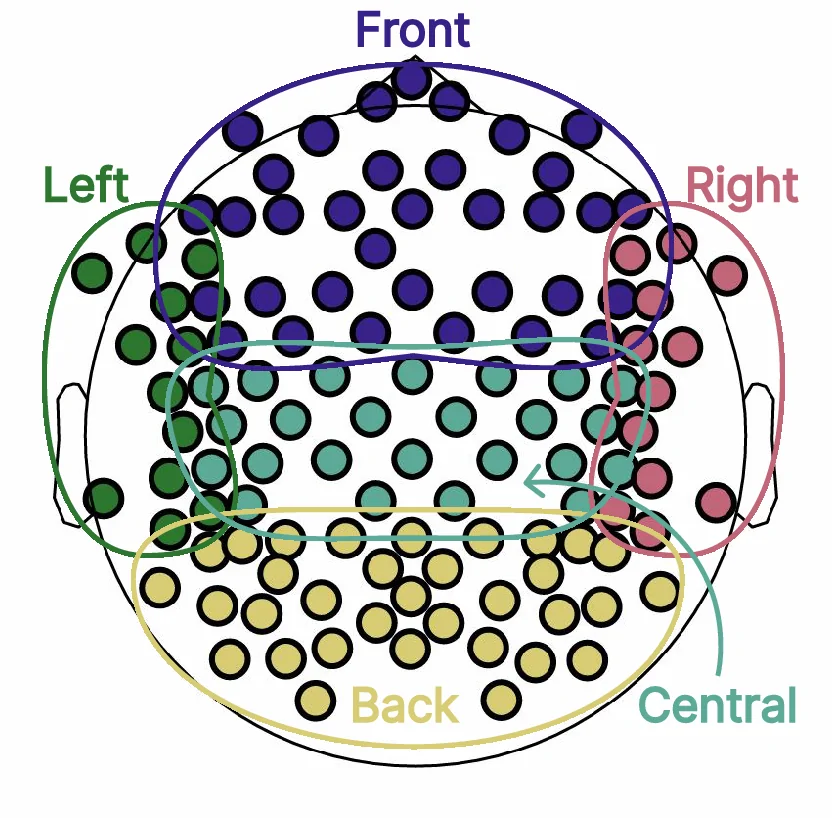
Regions-of-interest (ROIs) as used in the exploratory analysis. Electrodes were assigned to frontal (blue), left (green), central (turquoise), right (pink) or occipital (yellow) ROIs.

Given the exploratory nature and small sample size of this study, statistical adjustment for confounders was not feasible. As the measures differ spatially (see topographies in main article) we further performed a statistical analysis using the ROIs we defined (see Fig. 7). For each participant, condition, and EEG measure, amplitudes were averaged across all channels within each ROI to obtain region-specific values. Statistical comparisons were performed using paired t-tests between the three condition pairs. Effect sizes were quantified using Cohen’s d for paired samples, calculated as the mean difference divided by the standard deviation of the differences. Multiple comparisons correction was applied using the Benjamini-Hochberg false discovery rate procedure to control for the family-wise error rate across all ROI-measure-comparison combinations tested. Statistical significance was assessed at *α* = 0.05 after correction. We visualised the effect sizes as heatmaps which are reported below.

Statistical analyses were performed in Python using SciPy v1.15.3^60^ for paired t-tests and Bonferroni correction (Fig. S3, Table S2), statsmodels v0.14.4^61^ for Benjamini-Hochberg false discovery rate correction, and NumPy v2.2.5^62^ for effect size computation (calculated as the mean condition difference divided by the standard deviation of the differences, Fig. 3). Post-hoc statistical power in Table S2 was estimated using G*Power v3.1^63^.

## Supplementary Information

Supplementary Information.

## Data Availability

The de-identified participant data that support the findings of this study are available from the authors upon reasonable request and subject to institutional and ethical approvals.
