# Supplementary material for: An experimental study of the effect of neuromuscular blockade on EEG-based measures of awareness
Source: Sci Rep. 2026 May 2;16:29776. doi: 10.1038/s41598-026-50911-6 (PMC13612226; doi:10.1038/s41598-026-50911-6)
Supplement: Supplementary file 1 [file 41598_2026_50911_MOESM1_ESM.pdf]

Appendix: Supplemental data

1 Terminology

Terms such as “consciousness” and “awareness” have been used in various ways, sometimes causing confusion. Here we use the terms “conscious”/“consciousness” for the state/ability to experience anything, including dreaming, even when the subject is unable to respond. “Awareness” denotes here the ability to experience current sensory input from the body and world, but not dreaming, while “responsiveness” is the ability to respond verbally or non-verbally to stimuli[1]. Thus, a deeply anaesthetised patient can be “unaware” even if they are consciously experiencing a dream, while a paralysed patient can be both conscious and aware despite being unresponsive[1]. There are gray zones between these concepts, and further issues related to phenomenal consciousness vs. access consciousness[2], reportability, and different types of memory, etc.[3,4], but we will not address all these issues here.

With this in mind, to disentangle the three states that we compare in this study were named, we use the following short terms: (1) “awake-unparalysed” (responsive and aware; confirmed conscious by immediate conscious), (2) “awake-paralysed” (unresponsive (outside isolated forearm technique (IFT)) but aware; confirmed conscious by delayed report (or immediate during IFT)), and (3) “sedated-paralysed” (unresponsive and (presumably) unaware (and unconscious)). Note that neither unconsciousness nor unresponsiveness during sedated-paralysed can be objectively verified, but the sedation state was comparable to those typically used as ”unconscious control” in consciousness science. That said, when asked right after the experiment, all six participants reported remembering no conscious experience from the period of sedation (which included train-of-four testing supramaximal electrical stimulation of a peripheral nerve that is perceived as unpleasant, performed before administering the neuromuscular blocking agent reversal agent).

2 Investigation of artefact removal effects

2.1 Pre-processing

We refer to the data processed to this point as ’Raw’ in the context of the current analysis. In our attempts to reduce the impact of electromyogram (EMG) contamination[5] we investigated multiple approaches. (1) ICA: compute independent components using Infomax independent component analysis (ICA), see[6],

and label components using ICLabel[7] retaining only components labelled with 'brain' or 'other'. (2) ARICA: apply Autoreject automated artefact rejection[8], repair only without rejection, apply ICA as in (1) but only on epochs not marked for rejection, run Autoreject again with same parameters. (3) EMG: compute independent components using Picard[9] and labelled using the `find_bads_muscle` function in MNE. The parameters of the labelling method were fine tuned using the electroencephalogram (EEG) data in the current analysis, thus the approach may be considered as over-fitted for our analysis. And finally (4) CSDEMG: identical to (3) with the difference that we first transformed the data to current source density, see[10].

2.2 Impact of artefact removal

Removal of artefacts with ICA affected the awake-unparalysed state data more than the data from the awake-paralysed and sedated-paralysed state (see **Figure S1 A**). Bandpower decreased particularly in the peripheral electrodes in all canonical bands. The differences were small, also in the awake-unparalysed condition, and not significant in any band (see **Figure S1 B**). Overall, the difference in bandpower values in the gamma band between all three conditions decreased after artefact removal (see **Figure S1 C**).

In an explorative analysis we investigated further artefact removal methods and quantified their differences as changes in mean F_1 score at differentiating the aware (awake-unparalysed and awake-paralysed) and unaware (sedated-paralysed) states, see **Figure S2 (left)**. The line plot supports the the result of the statistical analysis as the differences in F_1 score between methods were quite small. None of the artefact removal methods were able to substantially affect the ability of the classifier to recognise the awake-paralysed state as aware. We investigated five feature types in more detail, see **Figure S2 (right)**. (1) The alpha peak frequency feature was not affected by the artefact removal but performance of this feature was generally low. (2) The slope feature performed nearly perfectly in the when the awake-paralysed state was excluded (naïve approach) but none of the artefact removal methods were able to alleviate the impact of including the awake-paralysed condition in the testing data. (3) Similarly, Lempel-Ziv complexity (LZc) features performed well in the naïve approach and did not improve after applying artefact removal. (4) Alpha bandpower features did not perform quite as well as (2) and (3) but performed the best after inclusion of paralysed-awake. (5) The outflow feature had the most variance in performance and was the feature to benefit the most from applying autoreject artefact removal.

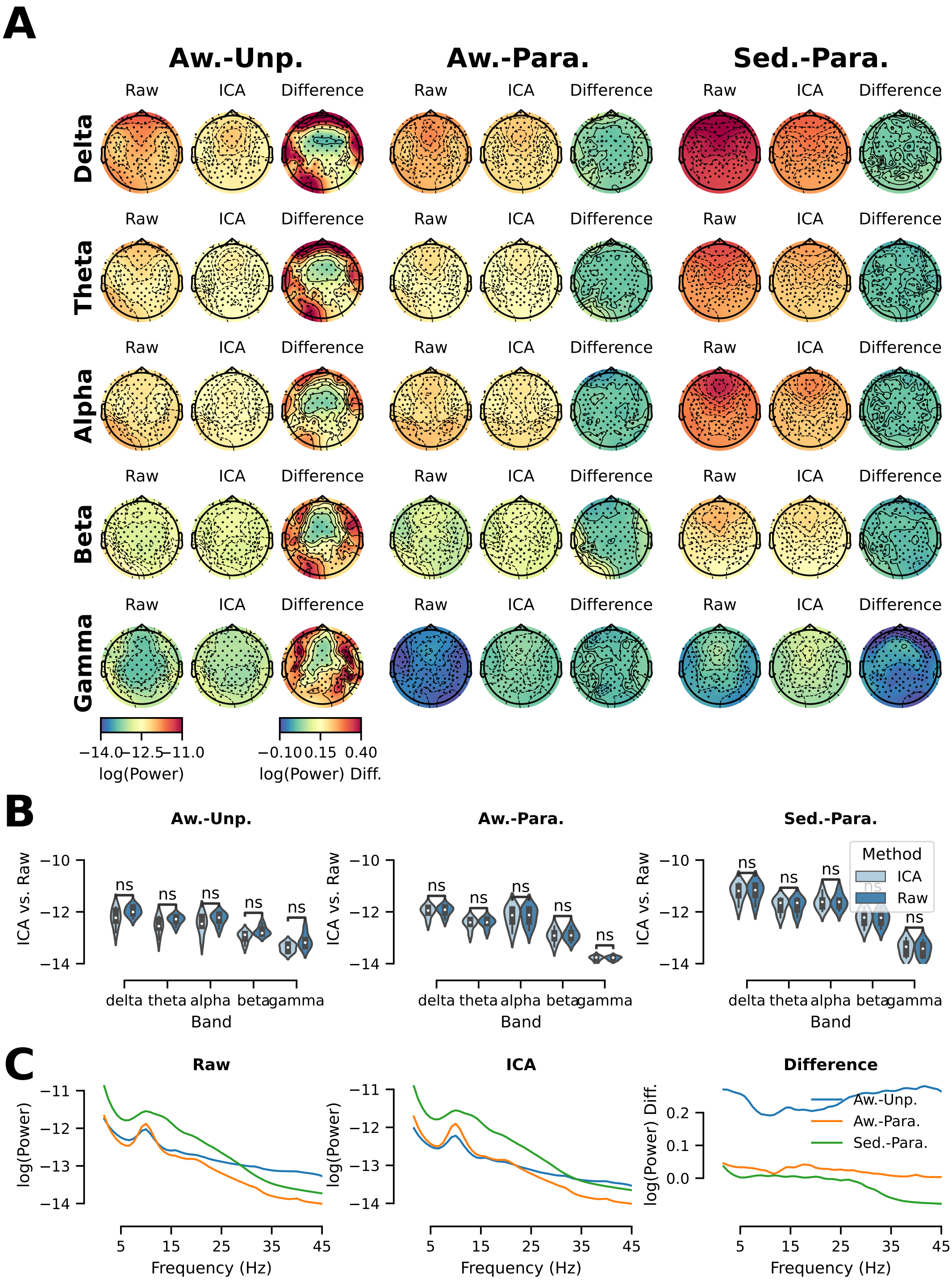

Figure S1 – (A) Power spectral density (PSD) values were visualised as topographies for each canonical frequency band (rows), for each condition (awake-unparalysed, awake-paralysed and sedated-paralysed; columns) and within each condition before artefact removal with ICA (Raw), after artefact removal (ICA) and as the difference between both (Difference). (B) The distribution of the PSD values was visualised as a group of violin plots for each condition. Colours indicate whether the values of the raw (dark blue) or the ICA (light blue) cleaned data were displayed. Statistical tests (paired t-test) were performed between raw and ICA values. Values were aggregated across channels and epochs resulting in one value per participant and condition (six values per condition). P-values were Bonferoni corrected for 15 comparisons (ns $p > .05$, * $p \leq .05$, ** $p \leq .01$, *** $p \leq .001$, **** $p \leq .0001$) (C) PSD visualised as line plots and averaged across all epochs and channels. Raw, ICA artefact removal and difference values were visualised individually using separate lines per condition.

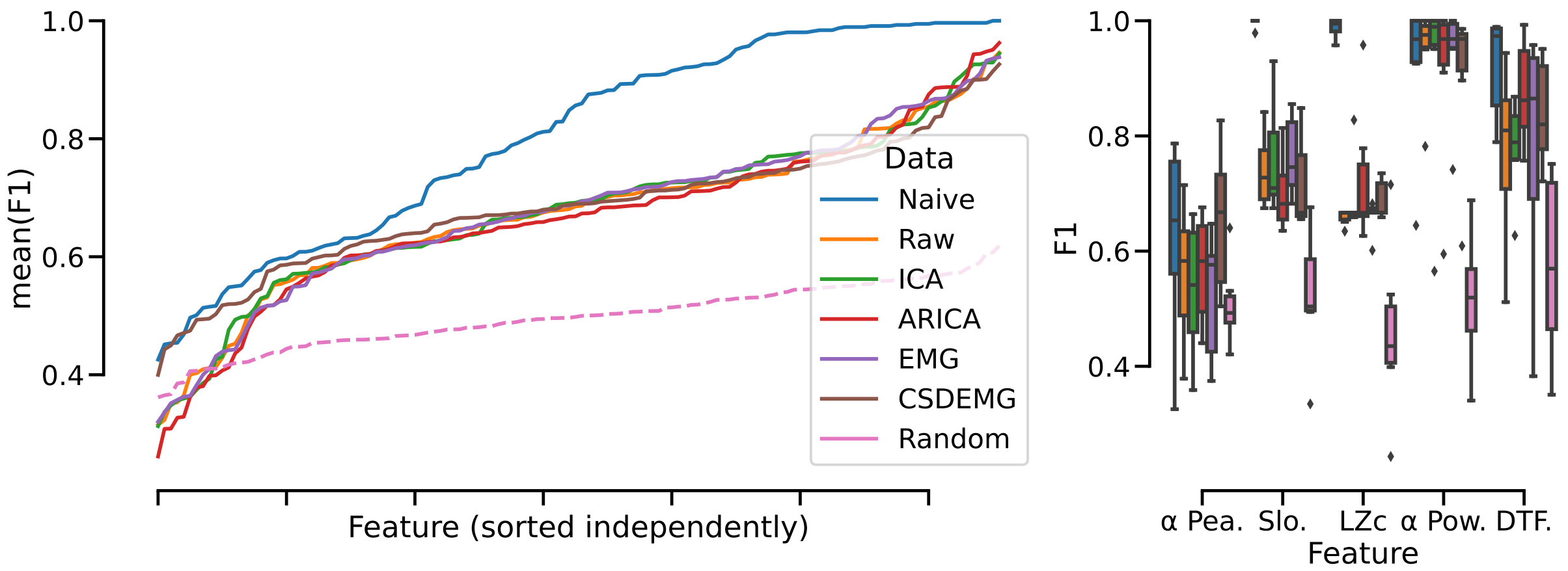

Figure S2 – Compares mean F_1 score of naive approach (no awake-paralysed data in testing or training), raw data (awake-paralysed used in testing, no artefact removal) and data after artefacts were removed with IC Label (ICA), IC Label + autoreject (ARICA), MNE EMG detection (EMG), transform to CSD and then use MNE EMG detection (CSDEMG). F_1 scores were further computed with randomised training labels and the raw features (dashed pink line). Scores on all lines were sorted by mean, thus the x-axis may not always be for the same feature. This is of particular importance when interpreting the F_1 scores with randomised labels as one cannot conclude that they correlate with the non-randomised scores. On the right hand side, we show the exemplary impact on F_1 score in a box plot for alpha peak frequency, slope (low+high), LZc, alpha log(power) and DTF (outflow). Colours of boxes correspond to lines in left plot.

2.3 Why did the ICA based artefact removal only have a small effect?

Artefact removal affected mostly the awake-unparalysed state data on peripheral electrodes. The main issue that we observed in our analysis was the misclassification of awake-paralysed as sedated-paralysed, but the EEG recorded during these states was not as contaminated with muscle artefacts. Thus, the classification of the awake-paralysed state as aware using the features tested was not meaningfully improved by automatic muscle artefact removal from the EEG (see **Figure S2**).

3 Individual differences alpha

We further explored the changes in alpha band power between conditions for each of the six participants individually, see **Figure S3**. For the statistical analysis we focused on the frontal region of interest (ROI). For all six participants, alpha power increased significantly between the awake-unparalysed and sedated-paralysed state. For three participants alpha power did not change, for two participants alpha increased and for one it decreased from awake-unparalysed to awake-paralysed state. For five of six participants alpha increased significantly from awake-paralysed to sedated-paralysed state.

For classification, the support vector classifier leverages the spatial pattern of alpha activity, which typically showed anteriorisation (frontal $>$ posterior power) under propofol sedation. This pattern was

dose-dependent: Participant 0, who received a smaller dose than the others, exhibited reduced anteriorisation (**Figure S3**). During leave-one-subject-out cross-validation, the classifier, trained on participants with stronger anteriorisation, struggled to distinguish P0's sedated-paralysed state from awake-paralysed. Consequently the F_1 score of participant 0 was the lowest with 0.8 (for reference P1: 0.95, P2: 0.99, P3: 0.99, P4: 1.00, P5: 0.95). This highlights a key limitation: alpha-based awareness detection assumes sufficient propofol dosing to induce the anteriorised alpha pattern characteristic of surgical anaesthesia. Future work should quantify dose-response relationships and explore whether spatial alpha patterns remain reliable at lower sedative doses

4 Assigning unique labels to all three states

In addition to the combinations of classes described in the main paper we also explored how well the classifier performed when we include awake-unparalysed, awake-paralysed and sedated-paralysed in training and testing with unique labels.

4.1 Training and testing

Using this approach we investigated whether there were consistent differences in between the measures computed from each of the three states. One should note that it would be difficult to implement this approach on a larger sample as awake-paralysed data would need to be collected without anaesthesia/sedation. When inspecting the confusion matrices in **Figure S4** it is interesting to note which measures lead to confusion between sedated-paralysed and awake-paralysed (higher errors in the top left, measure less likely to detect accidental awareness during general anaesthesia (AAGA)) and which to confusion between awake-paralysed and awake-unparalysed (higher errors on the bottom right, measure more likely to detect AAGA). One can also regard only the errors when classifying the awake-paralysed state. If the error for predicting awake-paralysed as sedated-paralysed is higher this is more problematic for predicting AAGA than if the error is high for predicting awake-paralysed as awake-unparalysed. The former is the case for LZc, slope (high), directed transfer function (outflow), delta, and gamma and the latter for slope (low), theta, alpha and beta. Again, predicting the state of the participant using alpha power led to the lowest error of awake-paralysed trials classified as sedated-paralysed (2%). The worst performance was again obtained using the alpha peak measure.

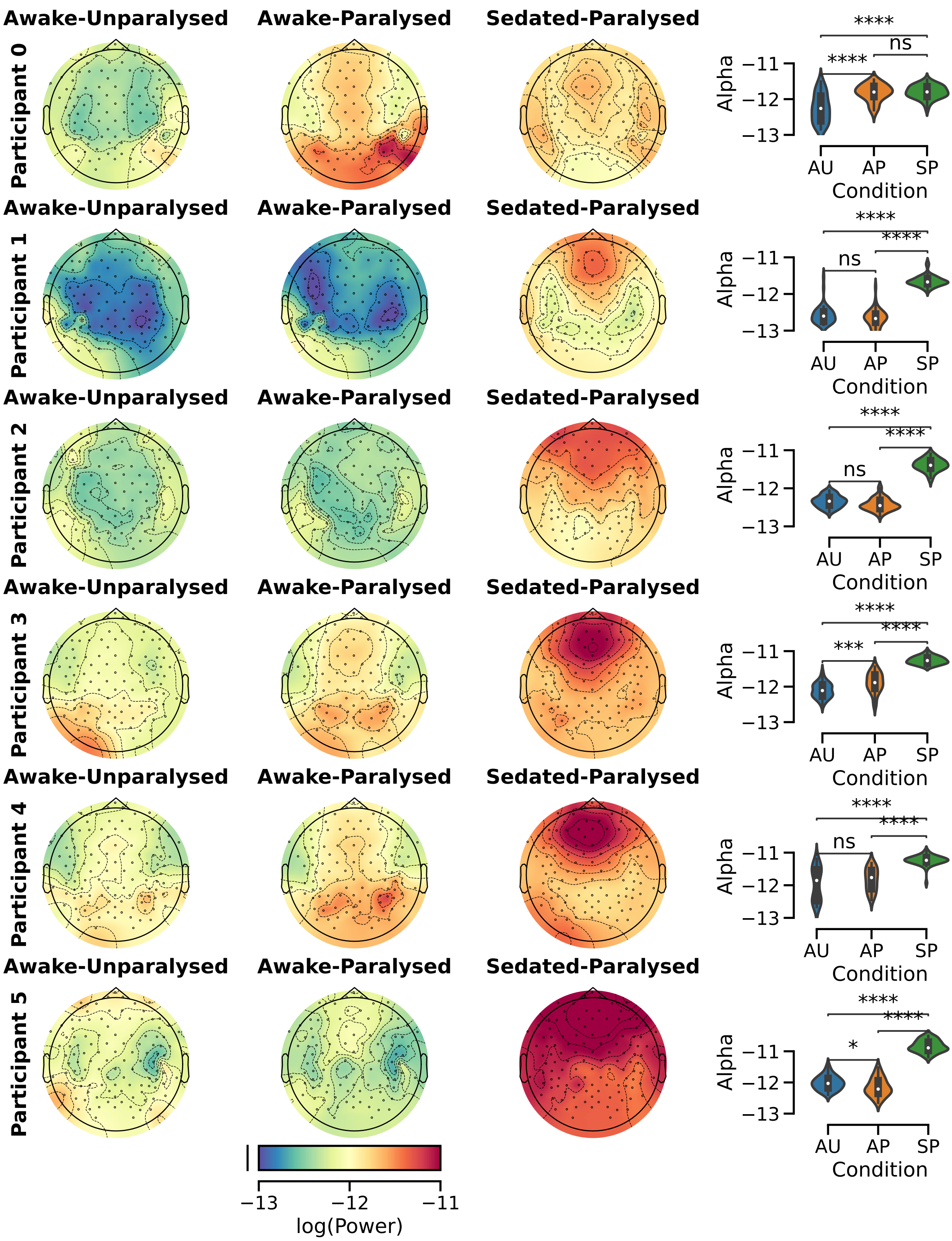

Figure S3 – Power spectral density (PSD) values (as log band power computed using fitting oscillations & one-over-F (FOOOF)) in the alpha band visualised as three topographies, one per condition (awake-unparalysed (AU), awake-paralysed (AP), sedated-paralysed (SP), per participant. Numerical distributions of PSD values were visualised as violin plots again split according to condition. Statistical tests (paired t-test) were performed between conditions. Values were aggregated across channels (using only the frontal region-of-interest) resulting in one value per epoch and condition (47 values per condition). P-values were Bonferroni corrected for 18 comparisons (ns $p > .05$, * $p \leq .05$, ** $p \leq .01$, *** $p \leq .001$, **** $p \leq .0001$).

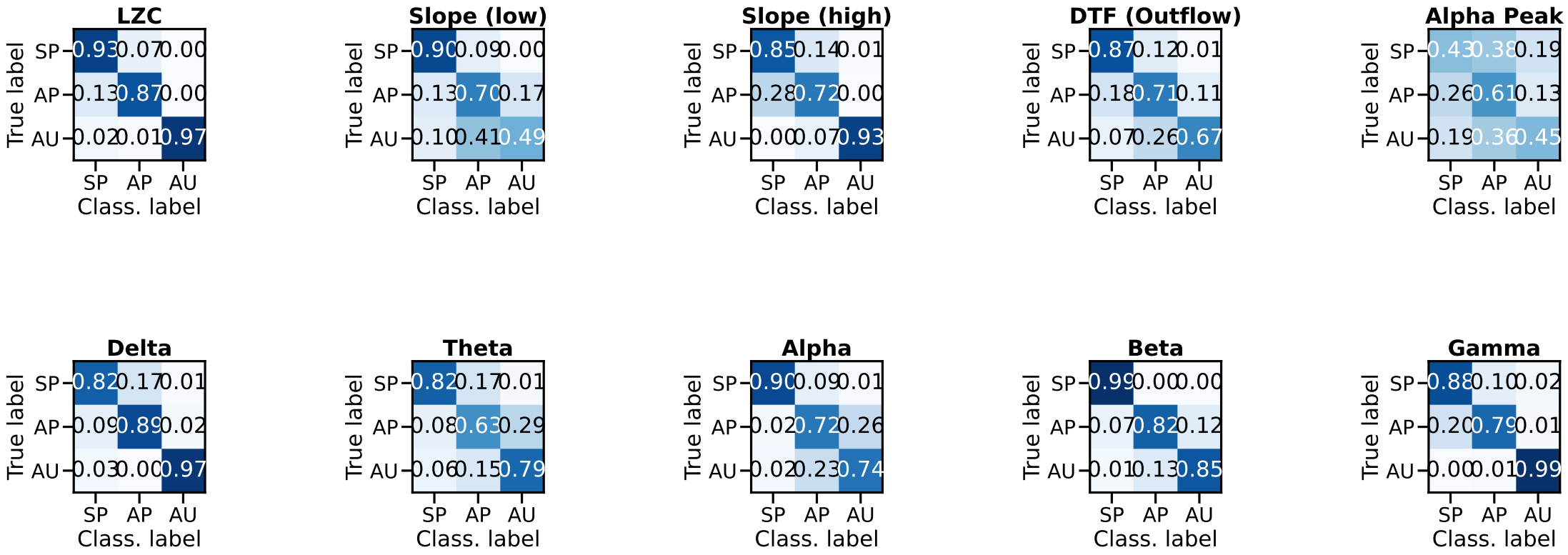

Figure S4 – Confusion matrices that were computed by classifying individual epochs in a leave-one participant out cross-validation scheme. The values reported in the matrix (AU: Awake-Unparalysed, AP: Awake-Paralysed, SP: Sedated-Paralysed) were computed as the mean across all cross-validation steps. All three states were included in training and testing. Please note that this training and testing approach could not be implemented with a larger sample as it is unlikely that the awake-paralysed state will be available. Results were computed using the measures indicated in the title of each matrix.

5 Exploratory analysis of features

Inspired by the selection of measures presented by Casey et al., 2024[11] we expanded our analysis to an exploration of a total of 55 features and compared their performance using the F_1 score computed by either training and testing the classifier on awake-unparalysed and sedated-paralysed epochs (naïve approach) or trained on awake-unparalysed and sedated-paralysed epochs and tested on awake-unparalysed, awake-paralysed and sedated-paralysed epochs (two class approach). All measures were computed scripts implemented in Python with the MNE toolbox[12]. Additional modules were used where indicated. *Spectral edge frequency 95 (SEF95)*: We computed the power spectral density (PSD) using Welch's method on the 1 to 45 Hz band[13–15]. We note that other studies used 1-55 Hz but this overlaps with line noise in many countries. We computed the cumulative trapezoid (using Scipy) over the PSD, normalised each value by the total, and determined the frequency at which the cumulative power crosses the 95th percentile. The log transformed values were used for classification with one value per channel (all channels) per epoch.

Absolute and relative band power:. As an alternative to our approach using FOOOF we also computed absolute and relative band power in all canonical frequency bands[16–18]. We computed the PSD using Welch's method on the 1-45 Hz band and extracted the mean for each frequency band and the mean of each band divided by the total power across all bands. This resulted in one value of absolute and relative power per canonical band, channel (all channels used) and epochs.

Alpha peak power and frequency: To align with the approach of previously published work we used Welch's method to compute the power in the Alpha band. We used NumPy to compute the maximum value per epoch and channel and stored peak power and frequency. We further computed the log ratio of Fz and Oz.

Frontal Alpha power: Computed the mean power in the alpha band over eight frontal channels (AFp1, AFp2, Fz, FC5, FC3, FCz, FC4, FC6) resulting in one value per epoch.

Spectral slope/exponent: As described above and we further computed one value per band.

FOOOF: As described above and we further computed the mean of the low and high band and combined both in one feature set. This then either resulted in one or two values per band, channel (all channels) and epochs.

LZc: As described above with the addition of computing the complexity with the epochs band pass filtered to the canonical bands. This resulted in one value per channel (all) and epoch.

Permutation Entropy: We computed permutation entropy[19, 20] using the AntroPy toolbox maintained by Raphael Vallat. We computed one value per band, channel (all) and epoch.

To compute the connectivity metrics we either used a set of eight frontal channels (AFp1, AFp2, Fz, FC5, FC3, FCz, FC4, FC6), eight occipital channels (P1, Pz, P2, PO7, PO8, O1, Oz, O2) or a distributed subset (F7, F3, Fz, F4, F8, T7, C3, Cz, C4, T8, P9, P7, P3, Pz, P4, P8, P10, O1, O2).

Frontal Alpha connectivity: Computed weighted phase lag index[21] in the alpha band between all pairs in the frontal channel set and averaged resulting in one value per epoch. *Delta-Alpha peak Coupling:* We filtered the epochs to the delta and alpha band, computed the Hilbert envelope of the alpha epochs and computed the correlation with the delta epochs[22].

Delta-Theta phase-amplitude coupling: We filtered the epochs to the high delta (2-4 Hz) and theta band, used Hilbert transformation to compute the phase of the delta epochs and the envelope of the theta epochs, assigned the phases to 18 bins, normalised each bin with the sum across all bins, computed the sum of each bin multiplied with the log of each bin and normalised with the one over the log of the number of bins[22].

DTF (Outflow): See above.

Casey et al., 2024 further investigated global and local efficiency[23] and normalised symbolic transfer entropy[24]. We decided to omit both these measures as they require optimisation of parameters on the dataset. This is not possible when attempting to classify awareness online. The parameters could be optimised on a separate subset of the data but we concluded this is outside the scope of our investigation.

6 Result Tables

Please refer to **Tables S1** and **S2** for group level values and statistics using the average of each measure across all channels. We provided classification accuracies for each feature set over the whole data set and for each condition separately in **Table S3**.

Table S1 – Measures of awareness in three conditions: (1) awake-unparalysed, (2) awake-paralysed, and (3) sedated-paralysed. Median values were computed across all epochs and electrodes for each participant which were then used to compute median, standard-deviation (SD) and range from minimum to maximum in the table below.

Measure	Statistic	awake-unparalysed	awake-paralysed	sedated-paralysed
LZc	Median	0.84	0.62	0.55
	SD	0.12	0.08	0.06
	Range	0.33 to 0.99	0.32 to 0.83	0.32 to 0.77
Spectral Slope (Low)	Median	-1.14	-0.95	-1.08
	SD	0.56	0.44	0.53
	Range	-4.67 to 0.83	-4.01 to 0.83	-3.13 to 0.89
Spectral Slope (High)	Median	-0.55	-4.02	-5.2
	SD	1.38	1.24	1.09
	Range	-6.69 to 3.32	-9.41 to 0.84	-10.39 to 3.61
Median Outflow	Median	-0.93	-0.79	-0.72
	SD	0.35	0.23	0.23
	Range	-2.25 to -0.11	-2.14 to -0.05	-2.22 to 0.0
Alpha Peak	Median	10.14	10.15	10.48
	SD	1.13	1.05	1.3
	Range	8 to 13	8.0 to 13.0	8 to 13
Delta Power	Median	-11.96	-11.95	-11.19
	SD	0.44	0.37	0.49
	Range	-13.12 to -8.31	-13.06 to -8.98	-12.7 to -6.79
Theta Power	Median	-12.3	-12.4	-11.73
	SD	0.34	0.32	0.38
	Range	-13.3 to -8.93	-13.4 to -9.81	-12.8 to 7.71
Alpha Power	Median	-12.67	-12.2	-11.63
	SD	0.5	0.58	0.43
	Range	-13.37 to -9.03	-13.61 to -9.73	-13.02 to -8.39
Beta Power	Median	-12.75	-12.92	-12.25
	SD	0.35	0.39	0.52
	Range	-13.73 to -8.9	-13.99 to -10.81	-13.93 to -8.65
Gamma Power	Median	-13.15	-13.79	-13.43
	SD	0.43	0.32	0.47
	Range	-14.14 to -9.12	-14.52 to -11.35	-14.69 to -8.78

Table S2 – Statistical comparisons between measures in the three different conditions: (1) awake-unparalysed (AU), (2) sedated-paralysed (SP) and (3) awake-paralysed (AP). Median values were computed across all epochs and electrodes for each participant which were then used to determine significance using Bonferoni corrected paired t-tests. Significant p-values are marked with a star (*). Cohen's *d* and statistical power were computed with median and SD from **Table S1** using G*Power 3.1[25]. For a measure to be considered successful, it should remain unchanged between awake-unparalysed and awake-paralysed states (AU vs AP), and differ when comparing either awake-unparalysed or awake-paralysed to the sedated-paralysed (AP vs SP). This criterion is highlighted in the statistical comparisons below. $^{-}$Measures with high power for AU vs AP shaded in red. $^{+}$ Measures with high power for distinguishing AP vs SP shaded in green.

**Measure**	**Statistic**	**AU vs SP**	**AU vs AP**	**AP vs SP**
LZc^{-}	t_5	10.08	6.62	2.12
	p	.001*	.007*	.526
	d	2.8	2.1	0.9
	$1-\beta$	.99	.98	.48
Spectral Slope (Low)	t_5	−0.98	−2.85	0.57
	p	1	.214	1
	d	0.1	0.4	0.2
	$1-\beta$	.05	.12	.07
Spectral Slope (High)$^{-}$	t_5	10.39	−4.73	5.18
	p	.001*	.031*	.021*
	d	3.7	2.6	1.0
	$1-\beta$	.99	.99	.51
DTF (Outflow)	t_5	−4.14	−2.97	−6.77
	p	.054	.188	.006*
	d	0.7	0.5	0.3
	$1-\beta$	.27	.15	.09
Alpha Peak	t_5	−0.79	−0.24	−0.82
	p	1	1	1
	d	0.3	0.0	0.3
	$1-\beta$	.09	.05	.09
Delta Power^{+}	t_5	−6.94	−0.35	−5.44
	p	.006*	1	.017*
	d	1.7	0.0	1.7
	$1-\beta$	.89	.05	.91
Theta Power^{+}	t_5	−6.53	4.24	−6.2
	p	.008*	.049*	.01*
	d	1.6	0.3	2.2
	$1-\beta$	.87	.09	.99
Alpha Power	t_5	−8.61	−0.93	−3.33
	p	.002*	1	.12
	d	2.2	0.9	1.1
	$1-\beta$	.99	.4	.58
Beta Power^{+}	t_5	−5.75	2.26	−6.88
	p	.013*	.442	.006*
	d	1.1	0.5	1.5
	$1-\beta$	.57	.19	.83
Gamma Power	t_5	3.09	5.86	−3.75
	p	.163	.012*	.08
	d	0.6	1.7	0.7
	$1-\beta$	.24	.89	.3

Table S3 – Classification accuracy with 95% Wilson score confidence intervals, derived from confusion matrices averaged per participant and aggregated across all six participants using leave-one-participant-out cross-validation (LOOCV; n = 6 per condition). F_1 scores are medians across LOOCV folds, consistent with Figure 7 (main article). Accuracy represents the proportion of epochs correctly classified per condition: SP epochs as unaware, and AP and AU epochs as aware. Confidence intervals were computed using the Wilson score method applied to participant-level aggregates. SP = Sedated-Paralysed; AP = Awake-Paralysed; AU = Awake-Unparalysed.

**EEG Measure**	F_1 **(2-class)**	F_1 **(naïve)**	**All Acc. (95% CI)**	**SP Acc. (95% CI)**	**AP Acc. (95% CI)**	**AU Acc. (95% CI)**
Abs. Pow. α	0.95	0.95	0.91 (0.67–0.97)	0.82 (0.30–0.90)	0.96 (0.44–0.97)	0.96 (0.44–0.97)
Abs. Pow. β	0.93	0.97	0.87 (0.61–0.94)	0.96 (0.44–0.97)	0.68 (0.30–0.90)	0.98 (0.44–0.97)
Abs. Pow. δ	0.90	0.87	0.88 (0.61–0.94)	0.79 (0.30–0.90)	0.94 (0.57–1.00)	0.91 (0.44–0.97)
Abs. Pow. F.	0.96	0.94	0.93 (0.67–0.97)	0.91 (0.44–0.97)	0.93 (0.57–1.00)	0.96 (0.44–0.97)
Abs. Pow. γ	0.65	0.97	0.61 (0.34–0.75)	1.00 (0.44–0.97)	0.01 (0.00–0.39)	0.82 (0.30–0.90)
Abs. Pow. H.	0.58	0.87	0.54 (0.29–0.71)	0.92 (0.44–0.97)	0.03 (0.00–0.39)	0.69 (0.30–0.90)
Abs. Pow. L.	0.95	0.92	0.92 (0.67–0.97)	0.87 (0.44–0.97)	0.96 (0.57–1.00)	0.95 (0.44–0.97)
Abs. Pow. θ	0.93	0.88	0.85 (0.61–0.94)	0.76 (0.38–0.96)	0.89 (0.44–0.97)	0.91 (0.57–1.00)
α Fz/Oz Ratio	0.92	0.93	0.89 (0.67–0.97)	0.90 (0.44–0.97)	0.88 (0.44–0.97)	0.89 (0.44–0.97)
α Peak Freq.	0.70	0.66	0.60 (0.34–0.75)	0.59 (0.19–0.81)	0.58 (0.19–0.81)	0.64 (0.19–0.81)
α Peak Power	0.93	0.91	0.89 (0.67–0.97)	0.83 (0.30–0.90)	0.92 (0.44–0.97)	0.94 (0.57–1.00)
δ-α Coupling	0.79	0.66	0.69 (0.44–0.84)	0.52 (0.19–0.81)	0.85 (0.57–1.00)	0.71 (0.30–0.90)
δ-θ PAC	0.79	0.65	0.66 (0.39–0.80)	0.07 (0.00–0.39)	0.98 (0.44–0.97)	0.91 (0.44–0.97)
FOOOF α	0.96	0.98	0.95 (0.74–0.99)	0.93 (0.44–0.97)	0.93 (0.44–0.97)	0.98 (0.44–0.97)
FOOOF β	0.76	0.99	0.76 (0.49–0.88)	0.96 (0.44–0.97)	0.33 (0.10–0.70)	0.99 (0.44–0.97)
FOOOF δ	0.88	0.91	0.82 (0.55–0.91)	0.85 (0.44–0.97)	0.71 (0.38–0.96)	0.89 (0.57–1.00)
FOOOF Full	0.67	0.99	0.74 (0.49–0.88)	0.99 (0.44–0.97)	0.23 (0.03–0.56)	1.00 (0.44–0.97)
FOOOF γ	0.67	1.00	0.66 (0.39–0.80)	0.96 (0.44–0.97)	0.01 (0.00–0.39)	1.00 (0.61–1.00)
FOOOF High	0.66	0.97	0.64 (0.39–0.80)	0.95 (0.44–0.97)	0.01 (0.00–0.39)	0.95 (0.44–0.97)
FOOOF L/H	0.99	0.99	0.95 (0.74–0.99)	0.99 (0.44–0.97)	0.89 (0.44–0.97)	0.95 (0.44–0.97)
FOOOF Low	0.99	0.99	0.93 (0.67–0.97)	0.99 (0.44–0.97)	0.87 (0.44–0.97)	0.94 (0.44–0.97)
FOOOF θ	0.92	0.92	0.86 (0.61–0.94)	0.76 (0.30–0.90)	0.88 (0.44–0.97)	0.94 (0.57–1.00)

Continued on next page

Table S3 – *Continued from previous page*

**EEG Measure**	F_1 **(2-class)**	F_1 **(naïve)**	**All Acc. (95% CI)**	**SP Acc. (95% CI)**	**AP Acc. (95% CI)**	**AU Acc. (95% CI)**
Front. α Con.	0.64	0.63	0.57 (0.34–0.75)	0.60 (0.19–0.81)	0.44 (0.10–0.70)	0.65 (0.19–0.81)
Front. α Pow.	0.85	0.77	0.77 (0.49–0.88)	0.60 (0.19–0.81)	0.82 (0.30–0.90)	0.90 (0.44–0.97)
LZC α	0.77	0.70	0.67 (0.39–0.80)	0.70 (0.30–0.90)	0.60 (0.19–0.81)	0.69 (0.30–0.90)
LZC β	0.94	0.98	0.86 (0.61–0.94)	0.98 (0.57–1.00)	0.64 (0.19–0.81)	0.95 (0.44–0.97)
LZC δ	0.73	0.66	0.64 (0.39–0.80)	0.58 (0.19–0.81)	0.69 (0.30–0.90)	0.66 (0.19–0.81)
LZC Full	0.67	0.99	0.66 (0.39–0.80)	1.00 (0.61–1.00)	0.00 (0.00–0.39)	0.98 (0.44–0.97)
LZC γ	0.86	1.00	0.85 (0.61–0.94)	0.99 (0.44–0.97)	0.55 (0.19–0.81)	1.00 (0.44–0.97)
LZC θ	0.60	0.58	0.55 (0.29–0.71)	0.61 (0.19–0.81)	0.50 (0.10–0.70)	0.56 (0.19–0.81)
SEF95	0.67	1.00	0.67 (0.39–0.80)	1.00 (0.61–1.00)	0.04 (0.00–0.39)	0.96 (0.44–0.97)
DTF (Outflow)	0.69	0.92	0.66 (0.39–0.80)	0.94 (0.57–1.00)	0.20 (0.03–0.56)	0.85 (0.44–0.97)
PE α	0.70	0.69	0.66 (0.39–0.80)	0.75 (0.30–0.90)	0.57 (0.19–0.81)	0.64 (0.19–0.81)
PE β	0.83	0.99	0.80 (0.55–0.91)	0.95 (0.44–0.97)	0.49 (0.10–0.70)	0.98 (0.44–0.97)
PE δ	0.70	0.62	0.64 (0.39–0.80)	0.63 (0.19–0.81)	0.69 (0.30–0.90)	0.61 (0.19–0.81)
PE Full	0.67	1.00	0.68 (0.44–0.84)	1.00 (0.61–1.00)	0.03 (0.00–0.39)	1.00 (0.61–1.00)
PE γ	0.70	1.00	0.71 (0.44–0.84)	1.00 (0.61–1.00)	0.13 (0.00–0.39)	1.00 (0.61–1.00)
PE θ	0.71	0.66	0.66 (0.39–0.80)	0.72 (0.30–0.90)	0.60 (0.19–0.81)	0.65 (0.19–0.81)
Rel. Power α	0.85	0.87	0.81 (0.55–0.91)	0.89 (0.44–0.97)	0.68 (0.38–0.96)	0.87 (0.44–0.97)
Rel. Power β	0.60	0.61	0.58 (0.34–0.75)	0.92 (0.44–0.97)	0.29 (0.03–0.56)	0.52 (0.19–0.81)
Rel. Power δ	0.82	0.84	0.77 (0.49–0.88)	0.92 (0.44–0.97)	0.63 (0.19–0.81)	0.77 (0.30–0.90)
Rel. Power Full	0.80	0.91	0.80 (0.55–0.91)	0.94 (0.44–0.97)	0.62 (0.19–0.81)	0.83 (0.30–0.90)
Rel. Power γ	0.67	0.99	0.68 (0.44–0.84)	1.00 (0.61–1.00)	0.08 (0.00–0.39)	0.96 (0.44–0.97)
Rel. Power H.	0.66	0.94	0.63 (0.39–0.80)	0.99 (0.44–0.97)	0.06 (0.00–0.43)	0.85 (0.44–0.97)
Rel. Power L.	0.77	0.90	0.76 (0.49–0.88)	0.94 (0.44–0.97)	0.56 (0.19–0.81)	0.77 (0.30–0.90)
Rel. Power θ	0.57	0.47	0.50 (0.25–0.66)	0.74 (0.30–0.90)	0.40 (0.10–0.70)	0.35 (0.10–0.70)
Spec. Exp. α	0.66	0.66	0.63 (0.39–0.80)	0.74 (0.30–0.90)	0.56 (0.19–0.81)	0.58 (0.19–0.81)
Spec. Exp. β	0.77	0.87	0.77 (0.49–0.88)	0.91 (0.44–0.97)	0.52 (0.19–0.81)	0.88 (0.44–0.97)

Continued on next page

Table S3 – *Continued from previous page*

EEG Measure	F_1 (2-class)	F_1 (naïve)	All Acc. (95% CI)	SP Acc. (95% CI)	AP Acc. (95% CI)	AU Acc. (95% CI)
Spec. Exp. δ	0.66	0.61	0.61 (0.39–0.80)	0.71 (0.30–0.90)	0.55 (0.19–0.81)	0.57 (0.19–0.81)
Spec. Exp. Full	0.69	0.99	0.72 (0.44–0.84)	0.98 (0.44–0.97)	0.19 (0.03–0.56)	0.98 (0.44–0.97)
Spec. Exp. γ	0.70	1.00	0.70 (0.44–0.84)	0.99 (0.44–0.97)	0.11 (0.00–0.39)	1.00 (0.44–0.97)
Spec. Exp. High	0.72	1.00	0.71 (0.44–0.84)	0.98 (0.44–0.97)	0.15 (0.00–0.43)	1.00 (0.61–1.00)
Spec. Exp. L/H	0.93	0.94	0.88 (0.61–0.94)	0.96 (0.44–0.97)	0.81 (0.30–0.90)	0.89 (0.44–0.97)
Spec. Exp. Low	0.93	0.94	0.88 (0.61–0.94)	0.96 (0.44–0.97)	0.80 (0.30–0.90)	0.89 (0.44–0.97)
Spec. Exp. θ	0.70	0.68	0.65 (0.39–0.80)	0.77 (0.30–0.90)	0.57 (0.19–0.81)	0.62 (0.19–0.81)

References

1. Sanders, R. D., Tononi, G., Laureys, S., Sleigh, J. W. & Warner, D. S. Unresponsiveness ≠ Unconsciousness. *Anesthesiology* **116,** 946–959. doi:10.1097/ALN.0b013e318249d0a7 (Apr. 2012).

2. Block, N. Phenomenal and Access Consciousness Ned Block and Cynthia MacDonald: Consciousness and Cognitive Access. *Proceedings of the Aristotelian Society* **108.** Publisher: [Aristotelian Society, Wiley], 289–317 (2008).

3. Lamme, V. A. F. How neuroscience will change our view on consciousness. *Cognitive Neuroscience* **1.** Publisher: Routledge _eprint: https://doi.org/10.1080/17588921003731586, 204–220. doi:10.1080/17588921003731586 (Aug. 2010).

4. Storm, J. F. *et al.* An integrative, multiscale view on neural theories of consciousness. English. *Neuron* **112.** Publisher: Elsevier, 1531–1552. doi:10.1016/j.neuron.2024.02.004 (May 2024).

5. Goncharova, I. I., McFarland, D. J., Vaughan, T. M. & Wolpaw, J. R. EMG contamination of EEG: spectral and topographical characteristics. *Clinical Neurophysiology* **114,** 1580–1593. doi:10.1016/S1388-2457(03)00093-2 (Sept. 2003).

6. Delorme, A., Sejnowski, T. & Makeig, S. Enhanced detection of artifacts in EEG data using higher-order statistics and independent component analysis. *NeuroImage* **34,** 1443–1449. doi:10.1016/j.neuroimage.2006.11.004 (Feb. 2007).

7. Pion-Tonachini, L., Kreutz-Delgado, K. & Makeig, S. ICLabel: An automated electroencephalographic independent component classifier, dataset, and website. *NeuroImage* **198,** 181–197. doi:10.1016/j.neuroimage.2019.05.026 (Sept. 2019).

8. Jas, M., Engemann, D. A., Bekhti, Y., Raimondo, F. & Gramfort, A. Autoreject: Automated artifact rejection for MEG and EEG data. *NeuroImage* **159,** 417–429. doi:10.1016/j.neuroimage.2017.06.030 (Oct. 2017).

9. Ablin, P., Cardoso, J.-F. & Gramfort, A. Faster Independent Component Analysis by Preconditioning With Hessian Approximations. *IEEE Transactions on Signal Processing* **66.** Conference Name: IEEE Transactions on Signal Processing, 4040–4049. doi:10.1109/TSP.2018.2844203 (Aug. 2018).

10. Perrin, F., Bertrand, O. & Pernier, J. Scalp Current Density Mapping: Value and Estimation from Potential Data. *IEEE Transactions on Biomedical Engineering* **BME-34.** Conference Name: IEEE Transactions on Biomedical Engineering, 283–288. doi:10.1109/TBME.1987.326089 (Apr. 1987).

11. Casey, C. P. *et al.* Evaluation of putative signatures of consciousness using specific definitions of responsiveness, connectedness, and consciousness. *British Journal of Anaesthesia* **132,** 300–311. doi:10.1016/j.bja.2023.09.031 (Feb. 2024).

12. Gramfort, A. *et al.* MEG and EEG data analysis with MNE-Python. English. *Frontiers in Neuroscience* **7.** Publisher: Frontiers. doi:10.3389/fnins.2013.00267 (Dec. 2013).

13. Nieuwenhuijs, D., Coleman, E. L., Douglas, N. J., Drummond, G. B. & Dahan, A. Bispectral Index Values and Spectral Edge Frequency at Different Stages of Physiologic Sleep. en-US. *Anesthesia & Analgesia* **94,** 125. doi:10.1213/00000539-200201000-00024 (Jan. 2002).

14. Sleigh, J. W., Andrzejowski, J., Steyn-Ross, A. & Steyn-Ross, M. The Bispectral Index: A Measure of Depth of Sleep? en-US. *Anesthesia & Analgesia* **88,** 659. doi:10.1213/00000539-199903000-00035 (Mar. 1999).

15. Bruhn, J. *et al.* Correlation of approximate entropy, bispectral index, and spectral edge frequency 95 (SEF95) with clinical signs of ”anesthetic depth” during coadministration of propofol and remifentanil. eng. *Anesthesiology* **98,** 621–627. doi:10.1097/00000542-200303000-00008 (Mar. 2003).

16. Purdon, P. L. *et al.* Electroencephalogram signatures of loss and recovery of consciousness from propofol. *Proceedings of the National Academy of Sciences* **110.** Publisher: Proceedings of the National Academy of Sciences, E1142–E1151. doi:10.1073/pnas.1221180110 (Mar. 2013).

17. Banks, M. I. *et al.* Cortical functional connectivity indexes arousal state during sleep and anesthesia. *NeuroImage* **211,** 116627. doi:10.1016/j.neuroimage.2020.116627 (May 2020).

18. Blain-Moraes, S. *et al.* Neurophysiological Correlates of Sevoflurane-induced Unconsciousness. *Anesthesiology* **122,** 307–316. doi:10.1097/ALN.0000000000000482 (Feb. 2015).

19. Liang, Z. *et al.* Permutation auto-mutual information of electroencephalogram in anesthesia. en. *Journal of Neural Engineering* **10.** Publisher: IOP Publishing, 026004. doi:10.1088/1741-2560/10/2/026004 (Feb. 2013).

20. Kreuzer, M. *et al.* Spectral and entropic features are altered by age in the electroencephalogram in patients under sevoflurane anesthesia. *Anesthesiology* **132,** 1003–1016. doi:10.1097/ALN.0000000000003182 (May 2020).

21. Vinck, M., Oostenveld, R., van Wingerden, M., Battaglia, F. & Pennartz, C. M. A. An improved index of phase-synchronization for electrophysiological data in the presence of volume-conduction, noise and sample-size bias. *NeuroImage* **55,** 1548–1565. doi:10.1016/j.neuroimage.2011.01.055 (Apr. 2011).

22. Tsai, F.-F., Fan, S.-Z., Cheng, H.-L. & Yeh, J.-R. Multi-timescale phase-amplitude couplings in transitions of anesthetic-induced unconsciousness. en. *Scientific Reports* **9.** Publisher: Nature Publishing Group, 7815. doi:10.1038/s41598-019-44238-8 (May 2019).

23. Blain-Moraes, S. *et al.* Network Efficiency and Posterior Alpha Patterns Are Markers of Recovery from General Anesthesia: A High-Density Electroencephalography Study in Healthy Volunteers. English. *Frontiers in Human Neuroscience* **11.** Publisher: Frontiers. doi:10.3389/fnhum.2017.00328 (June 2017).

24. Lee, U. *et al.* Disruption of Frontal-Parietal Communication by Ketamine, Propofol, and Sevoflurane. *Anesthesiology* **118,** 1264–1275. doi:10.1097/ALN.0b013e31829103f5 (June 2013).

25. Faul, F., Erdfelder, E., Lang, A.-G. & Buchner, A. G*Power 3: A flexible statistical power analysis program for the social, behavioral, and biomedical sciences. en. *Behavior Research Methods* **39,** 175–191. doi:10.3758/BF03193146 (May 2007).
